# Dapagliflozin vs empagliflozin in patients with chronic heart failure: a registry analysis

**DOI:** 10.3325/cmj.2025.66.135

**Published:** 2025-04

**Authors:** Ivana Jurin, Irzal Hadžibegović, Hrvoje Jurin, Diana Rudan, Nikola Pavlović, Marija Radić, Šime Manola, Vladimir Trkulja

**Affiliations:** Jurin et al: Dapagliflozin and empagliflozin for heart failure; 1 Department of Cardiovascular Diseases, University Hospital Dubrava, Zagreb, Croatia; 2 Faculty of Dental Medicine and Health Care, Josip Juraj Strossmayer University, Osijek, Croatia; 3 Department of Cardiovascular Diseases, University Hospital Centre Zagreb, Zagreb, Croatia; 4 University North, Koprivnica, Croatia; 5 Zagreb University School of Medicine, Zagreb, Croatia; 6 Department of Pharmacology and Clinical Pharmacology, Zagreb University School of Medicine, Zagreb, Croatia

## Abstract

**Aim:**

To assess the relative efficacy of dapagliflozin and empagliflozin in routinely treated chronic heart failure (CHF) patients.

**Methods:**

Data from a registry of prevalent and incident CHF patients were used to set up cohorts (new-user design) of patients started on dapagliflozin or empagliflozin in addition to other guideline-directed therapy. Cohorts were mutually balanced on a range of characteristics, and were assessed for the incidence of a composite of all-cause death/major adverse cardiac events (primary outcome) over the initial 6 months of treatment, and for New York Heart Association (NYHA) functional class at 6 months (secondary outcome). Frequentist and Bayes estimates were generated for the dapagliflozin vs empagliflozin comparison.

**Results:**

In both prevalent (dapagliflozin n = 393, empagliflozin n = 328) and incident (dapagliflozin n = 124, empagliflozin n = 116) patients, those prescribed dapagliflozin had somewhat higher incidence of the primary outcome, but the confidence intervals were wide (RR = 1.385, 95%CI 0.882-2.173 [prevalent], RR = 2.192, 95%CI 0.765-6.282 [incident]), and were more likely to present with a worse NYHA class at 6 months (OR = 1.552, 95%CI 1.142-2.108 [prevalent], OR = 1.503, 95%CI 0.844-2.676 [incident]). In the pooled data, primary events (n = 102) were more common in dapagliflozin-prescribed patients (frequentist estimate RR = 1.519, 95%CI 1.239-1.861; Bayes RR = 1.380, 95%CrI 0.981-1.944). Dapagliflozin-prescribed patients were also more likely to have a worse NYHA class at 6 months (OR = 1.540, 95%CI 1.208-1.962; Bayes OR = 1.425, 95%CrI 1.098-1.781).

**Conclusion:**

CHF patients prescribed with dapagliflozin had poorer outcomes than their empagliflozin-prescribed peers over the initial 6 months of treatment. Data emphasize a need for a direct randomized comparison of the two treatments in this setting.

The current guidelines of the European Society of Cardiology (ESC) (1) for the management of patients with heart failure (HF) denote two sodium-glucose co-transporter 2 inhibitors (SGLT2i) – dapagliflozin and empagliflozin – as the only pharmacological options with a disease-modifying effect in chronic heart failure (CHF) across the entire range of left ventricular ejection fraction (LVEF) values. Hence, it is strongly recommended (1) that patients with reduced (HFrEF, LVEF≤40%), mildly reduced (HFmrEF, LVEF 41%-49%), or preserved (HFpEF, LVEF≥50%) ejection fraction should receive these treatments.

A serendipitous observation of cardiac benefits in type 2 diabetes (T2DM) patients treated with dapagliflozin or empagliflozin (2,3), and subsequent pivotal trials specifically in patients with CHF (4-7) boosted extensive mechanistic research. Although many elements might not have been fully elucidated, it is obvious that cardiovascular and renal benefits of SGLT2 inhibition arise from a multitude of (sub)cellular effects in the kidney, heart, and other tissues where fundamental biological processes (eg, metabolic and ionic homeostasis, cell-cycle regulation, apoptosis, autophagy, energetic metabolism, and mitochondrial function) are affected, resulting in organ/system changes like increased blood oxygen-carrying capacity, reduced tissue fat, improved glucose control, increased diuresis, reduced intravascular volume, improved endothelial function, improved ventricular loading conditions and work efficiency, and many others, eventually resulting in improved cardiovascular and renal health (8,9). Currently, it is implicitly understood that the reported effects/mechanisms are due to the concept of “SGTL2 inhibition;” however, at least some effects might not be comparably intense for different compounds of the gliflozin class (10). In a clinical sense, and specifically in the CHF setting, the two approved gliflozins (dapagliflozin, empagliflozin) yield comparable benefits, with comparable safety profiles [pivotal clinical trials (4-7), and *post-hoc* evaluations (11,12)], hence neither drug is preferred by the ESC guidelines (1). Still, based on small differences in the effects between dapagliflozin and empagliflozin (vs placebo) in pivotal trials, it has been suggested that in some health care systems cost-effectiveness of dapagliflozin could be somewhat more favorable than that of empagliflozin (13,14). On the other hand, a recent pharmacoepidemiological study suggested a 10% lower relative risk (RR) of hospitalization (95%CI 6% to 14%) and a 9% lower risk of all-cause death (95%CI 0 to 18%) within 1 year in patients prescribed with empagliflozin vs those prescribed with dapagliflozin (15). Considering that the study was based on administrative data, the suggested mild benefit in favor of empagliflozin should be viewed with caution (15).

We aimed to assess the relative efficacy of dapagliflozin vs empaglifozin in a cohort of routinely treated CHF patients. Based on the pivotal trial data (4-7,11,12), we expected that the two treatments would be comparable in this respect, but we did not intend to test any formal hypothesis. Rather, we considered it reasonable to rely upon numerical trends/effect estimates as supportive or opposing to this assumption.

## Patients and methods

### Study outline

The present analysis is based on data routinely collected in a registry of CHF patients maintained at the Dubrava University Hospital, Zagreb, Croatia (the CaRD registry, NCT06090591), and was approved by the Institutional Ethics Committee (2022/1403-01). In line with the Institution’s teaching status, all patients consented to the use of their anonymized data for the research and publishing purposes. Eligible for the analysis were patients started on dapagliflozin or empagliflozin between January 1, 2022 and July 31, 2023. Owing to the timing of regulatory approvals of the two drugs in treatment of CHF, in some patients SGLT2i were started after the commencement of other CHF treatments, ie, with a lag of a minimum 1 month and up to 10 months (prevalent patients), whereas in others SGLT2i were started together with other CHF treatments, ie, immediately upon the CHF diagnosis (incident patients). In the first step, these two patient subsets were analyzed separately (referred to as “Study 1” [prevalent patients] and Study 2 [incident patients]). Since the estimates generated in Study 1 and Study 2 were reasonably similar, data were merged and analyzed jointly (prevalent + incident patients). Finally, we explored potential modification of the dapagliflozin vs empagliflozin outcomes by chronic kidney disease (CKD or preserved renal function), and by the baseline LVEF (HFrEF or HFm/pEF).

### Patients and patient management

The CaRD registry was set up to capture adult CHF patients (≥18 years of age) treated with the new generation of disease-modifying treatments. The present analysis focused on patients started on SGLT2i specifically for the treatment of CHF, in line with the ESC recommendations (1), between January 1 2022 and July 31, 2023. The patients were included if they (i) had a relatively shorter history of CHF at the commencement of SGLT2i (ie, ≤18 months) to avoid potential confounding arising from possible imbalances in the burden of previous disease duration or other guideline-directed medical therapy (GDMT); and (ii) were free of conditions that could have affected life expectancy/evolution of CHF but might not have been controllable in the data analysis (ie, active malignant disease, on-going or recent anti-cancer treatment, HIV infection or pharmacological immunosuppression [transplant recipients, systemic inflammatory diseases]).

The registry included patients managed by five comparably experienced cardiologists specialized in heart failure. All diagnostic work-up and criteria, follow-up, and pharmacological and non-pharmacological treatments were implemented in line with the actual ESC guidelines (1,16). Medical histories, comorbidities, and treatments were regularly updated. Heart failure was verified based on the functional status with New York Heart Association (NYHA) classification, echocardiographic assessments and estimated LVEF, and serum levels of the N-terminal fragment of the brain natriuretic peptide (NT-proBNP) (>600 pg/mL if with ongoing atrial flutter or fibrillation, >300 pg/mL otherwise). Other diagnostic work-up (eg, electrophysiological, laboratory, imaging) was steered by the managing cardiologist, in line with the individual patient particulars. The treatment of specifically CHF followed the guidelines (1,16): (i) in patients with HFrEF, disease-modifying treatments were introduced and dosed on the discretion of the managing cardiologist in line with the patients’ status, response to therapy, or possible contraindications; (ii) since in patients with HFmrEF or HFpEF these compounds are not considered effective (1,16), their use was based on the managing cardiologist discretion, and largely determined by comorbidities; (iii) implantable cardiac defibrillator devices were recommended to all patients with HFrEF of ischemic etiology and LVEF≤35% despite at least 3 months of GDMT treatment, and were considered in patients with HFrEF of non-ischemic etiology under the same conditions; (iv) diuretics were used in line with the recommendations (1,16). With respect specifically to the use of SGLT2i: (i) the decision to commence treatment, the choice of a specific compound and posology were based on the managing cardiologist’s discretion, in line with the recommendations (1,16), the individual patient’s characteristics, and the approved product labels (contraindications, special warnings, and precautions to use) (17,18); (ii) at the start of treatment, the information on demographics, ongoing treatments, comorbidities, and medical histories was updated (particularly, possible hospitalizations or emergency department visits for HF, cardio- or neurovascular incidents, and occurrence of urinary tract infections over the preceding 12 months); renal function (estimated glomerular filtration rate, eGFR, by the CKD-EPI 2021 equation), LVEF and NYHA class were reassessed, and NT-proBN*P* values were again measured. Registry data were updated through routine or unscheduled patient visits, on the occasion of emergency department visits or hospitalizations for heart failure, and through periodic checks of the hospital information system to capture other visits/hospitalizations (for any reason). Regarding specifically the use of SGLT2i as newly approved treatments in this indication, the time of their commencement was defined as “baseline” (new-user design). The subsequent visits were scheduled at varying time intervals, depending on the individual patient particulars, but all were scheduled for a control visit at 6 months after the start of treatment, as a standard routine procedure. Patients were reminded about the scheduled control visit (telephone, e-mail), typically around 10 days before the scheduled visit, or at the due date (if skipped) at the latest. The control visit included i) update of medical histories, ii) clinical evaluation and NYHA class, iii) LVEF assessment, iv) NT-proBNP measurement, v) eGFR re-estimation, vi) inquiries about possible intercurrent events, particularly in case of identified “other visits” or interventions not directly related to CHF.

### Outcomes

The study period was limited to 6 months to minimize the interference of intercurrent events (particularly possible end of treatment or switching between treatments). The primary outcome was a composite of all-cause mortality and major adverse cardiovascular events (MACE) during this period, and was preferred to avoid bias arising from competing events. We preferred all-cause over cause-specific mortality to avoid misclassification bias arising from uncertainty in the detection of the actual causes of death that may occur in a “real-life” setting, particularly in the case of out-of-the hospital deaths. For in-hospital deaths, events and timing were identified directly, whereas out-of-the hospital deaths were assessed through the contacts with the next of kin and death certificates. MACE was defined as follows: (i) CHF deterioration – hospitalization or emergency department visit for HF; ambulatory visits outside the scheduled visits due to symptom intensification; (ii) acute myocardial infarction/acute coronary syndrome; (iii) cerebrovascular incidents/transitory ischemic attack; (iv) pulmonary embolism; (v) deep vein thrombosis. MACE events/timing were ascertained directly or through hospital information system. The incidence of MACE was determined as a proportion of patients with at least one MACE (only a few repeated events were observed). The secondary outcome was NYHA class at the visit at 6 months. To avoid selection bias, we assigned NYHA class 5 to patients who died during the study period; assuming that the probability of death could have been affected by the assigned SGLT2i, the analysis of only survivors could have been biased, since they would have been selected by the less severe disease, and a treatment with a higher mortality would have had better NYHA class results. Other outcomes were recorded only in survivors (at the control visit): (i) NT-proBNP levels; (ii) LVEF; and (iii) eGFR.

### Statistical considerations

We did not calculate the sample size but deemed that the data at hand could still provide a meaningful insight into relative efficacy of the two SGLT2i: i) the included patients displayed a wide range of LVEF values; hence, we expected the incidence of the primary outcome in the range of 10%-15% over the observed 6 months, and expected it to be fairly comparable in the two treatment arms; ii) in Study 1 (prevalent patients), and overall, this would have meant a number of events enabling rather robust proportions, where a few events more or less occurring by chance in either arm would not have altered the relationship between the two treatments substantially.

We used optimization-based weighting (19) to balance the covariates between the dapaglifloizin and empagliflozin-treated patients, separately in Study 1 (prevalent) and Study 2 (incident) with average treatment effect (ATE) as the estimand. The method is based on the convex optimization problem, and finds the weights of minimum variance that balance the empirical distribution of the observed covariates to the prespecified levels (19). It was implemented in package *optweight* (20) in R (21), and standardized mean differences <0.1 indicated adequate balance. A total of 41 covariates (potential confounders) were addressed, including demographics, CHF characteristics (NYHA class, LVEF values, NT-proBNP levels, CHF-related events over the preceding 12 months), comorbidities, and concomitant pharmacological and non-pharmacological treatments. There were no missing data on any of the considered covariates. Primary analysis of the primary and secondary outcomes was done separately in Study 1 and Study 2. Secondary analysis considered joint data (i) as if coming from a single study (“naïve” approach), (ii) as a fixed-effect one-stage individual-patient data meta-analysis, and (iii) as a mixed-effects meta-analysis (random study effect). To explore effect modification by CKD and LVEF, dapagliflozin- and empagliflozin-treated patients (prevalent + incident) were balanced on the same covariates separately in patients with and without CKD, and those with HFrEF and those with HFm/pEF. The analytical model included an interaction term between treatment and CKD or LVE category, so that dapagliflozin vs empagliflozin estimates could be generated in each subset separately.

Weighted regression models were fitted to (i) probability of the primary outcome (log-binomial); (ii) probability of having a more severe NYHA class (cumulative logit). The model for NYHA included baseline NYHA as a covariate. All models employed robust (sandwich) variance estimation and Gauss-Hermite quadrature approximation. All analyses were repeated by fitting Bayesian models (except for the effect modification analysis) since they are generally more conservative, and we used a moderately informed skeptical normal prior for ln(RR)/ln(OR) centered at 0.0 [N(0, 0.355)] (assigns 95% probability to RR or OR between 0.5 and 2.0) – in line with the assumption of comparable treatment outcomes. The analysis was done on the intent-to-treat basis (in agreement with the “treatment policy estimand”) (22): patients were considered as dapagliflozin- or empagliflozin-treated based on the initially assigned treatment. Other outcomes are summarized by treatment. We used SAS 9.4 for Windows (SAS Inc., Cary, NC) *proc glimmix* and *proc genmod*, and package *brms* (23) in R.

### Sensitivity analysis

Having in mind residual confounding as an inherent problem of observational studies, we assumed that the present outcomes – regardless of whether suggesting similarity or differences between treatments – would be biased, either toward or away from the null. Therefore, we conceived a hypothetical strong biasing effect (RR = 1.40 regarding the primary outcome; OR = 1.50 regarding the NYHA class) disfavoring one or the other treatment (overall prevalence 35%, with an imbalance of 1.5:1.0 in dapagliflozin-treated vs empagliflozin treated patients, or *vice-versa*), and planned to correct the observed effect estimates for this biasing effect (24) [package *episensr* (25) in R]. In case of difference between treatments, we also calculated the E-value - the size of the confounding effect (on the relative risk scale) needed to explain away the observed treatment differences (26).

## Results

### Patient eligibility and characteristics

**Figure 1:**
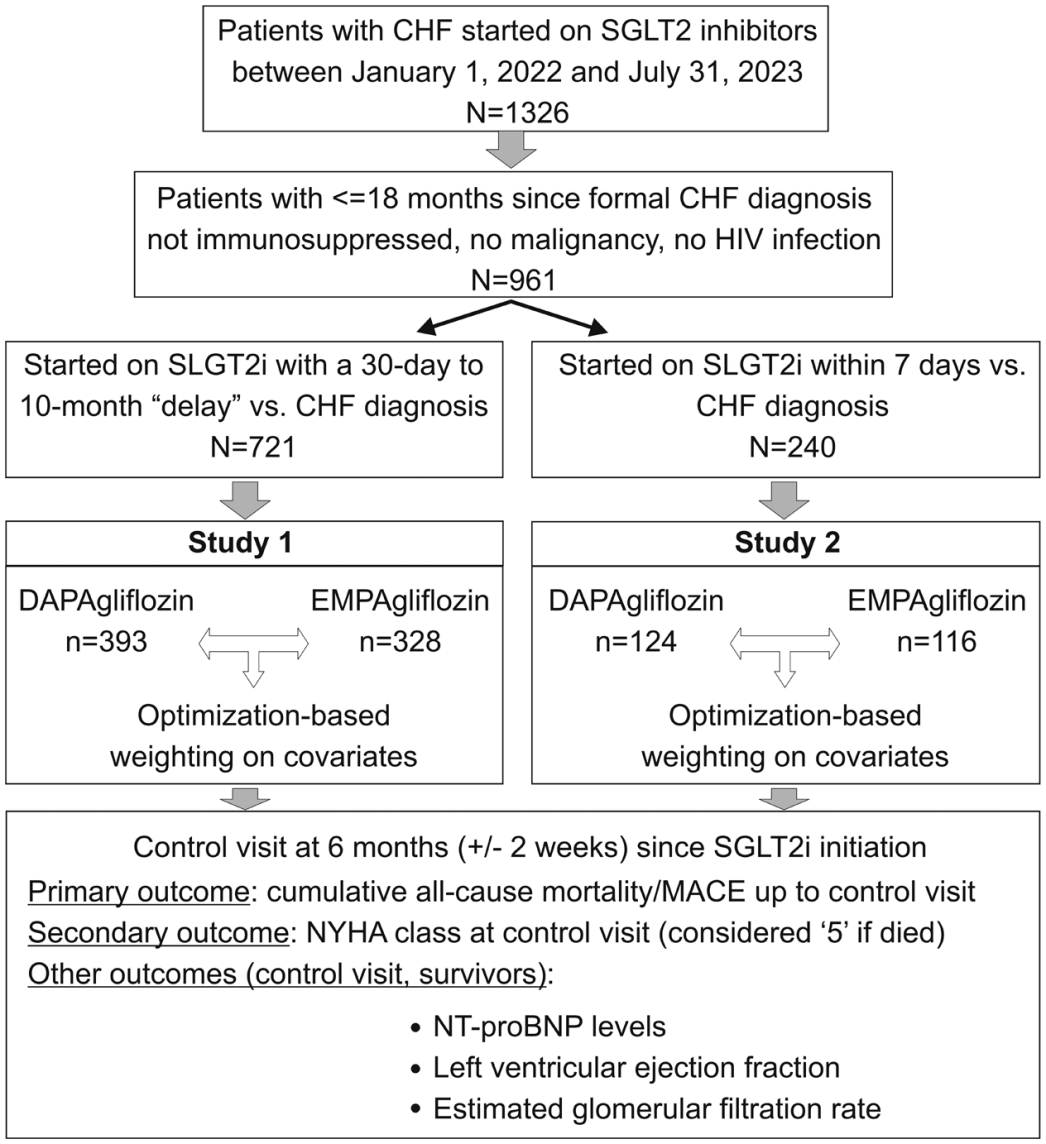
Outline of the present analysis. Included were chronic heart failure (CHF) patients embraced in an institutional registry, started on sodium-glucose co-transporter type 2 inhibitors (SGLT2i) dapagliflozin or empagliflozin, either with a delay vs the CHF diagnosis (prevalent patients, Study 1), or immediately after the CHF diagnosis (incident patients, Study 2). At the start of SGLT2i treatment (baseline), patients prescribed the two SGLT2i were mutually balanced (by optimization-based weighting) on a number of covariates, and were assessed at a control visit 6 months later. MACE – major adverse cardiovascular events; NT-proBNP – N-terminal pro-brain natriuretic polypeptide; NYHA – New York Heart Association.

A total of 961 patients were eligible for the analysis, mostly in Study 1(Figure 1), with comparable numbers prescribed with dapagliflozin (393 in Study 1, 126 in Study 2) and empagliflozin (328 in Study 1, 114 in Study 2) (Figure 1). Before weighting, dapagliflozin- and empagliflozin-prescribed patients differed regarding a number of characteristics: i) in Study 1 (prevalent patients) (Table 1), particularly regarding the prevalence of HFpEF/HFmEF/HFrEF, average LVEF, prevalence of chronic kidney disease, and average eGFR (d ≥0.300) (Table 1); (ii) in Study 2 (incident patients) (Table 2), particularly regarding the prevalence of LVEF≤40% and average LVEF, NYHA class, NT-proBNP concentration, prevalence of dyslipidemia, atrial fibrillation, coronary artery disease, hospitalization for HF as the occasion for CHF diagnosis, and the initiated treatment with mineralocorticoid receptor antagonists, diuretics, and antiplatelets (d ≥0.300) (Table 2). However, weighting enabled a fully adequate balance on all covariates in both studies: all d <0.1 (Table 1, Table 2).

**Table 1:** Patient characteristics in Study 1 by sodium-glucose transporter 2 inhibitor (SGLT2i) – dapagliflozin (DAPA) or empagliflozin (EMPA) – before and after optimization weighting^*†‡^

	Before weighting			After weighting		
	DAPA	EMPA	d	DAPA	EMPA	d
N	393	328	—	393	328	—
Age (years)	69 ± 11 (29-90)	69 ± 11 (27-90)	0.033	69 ± 11	69 ± 11	0.030
Male	260 (66.2)	216 (65.8)	0.006	65.6	67.5	-0.041
HF p EF (LVEF 50%-78%)	85 (21.6)	110 (33.5)	-0.269	27.0	27.0	0.000
HF mr EF (LVEF 41%-49%)	54 (13.7)	56 (17.1)	-0.092	15.3	15.3	0.000
HF r EF (LVEF≤40%)	254 (64.6)	162 (49.4)	0.312	57.7	57.7	0.000
LVEF (%)	38.5 ± 12.4 (15-78)	42.7 ± 11.8 (19-75)	-0.348	40.3 ± 12.7	40.6 ± 11.6	0.030
NYHA 1	22 (5.6)	20 (6.1)	-0.021	6.5	4.8	0.069
NYHA 2	188 (47.8)	177 (54.0)	-0.123	49.9	51.0	-0.020
NYHA 3	159 (40.5)	117 (35.7)	0.099	38.7	38.9	-0.004
NYHA 4	24 (6.1)	14 (4.3)	0.083	4.9	5.3	-0.018
NT-proBNP (pg/mL)	2530 (48-35000)	1798 (55-35000)	—	2237	2323	—
Ln(NT-proBNP)	7.859 ± 1.303	7.579 ± 1.205	0.223	7.713 ± 1.288	7.750 ± 1.216	-0.030
BMI (kg/m^2^)	28.4 (16.8-65.1)	29.6 (16.7-53.0)	—	28.7	29.4	—
Ln(BMI)	3.362 ± 0.177	3.398 ± 0.190	-0.194	3.357 ± 0.181	3.381 ± 0.187	-0.030
Chronic kidney disease	199 (50.6)	108 (32.9)	0.365	42.6	42.6	0.000
eGFR (mL/min/1.73m^2^)	59.9 ± 24.1	67.6 ± 20.4	-0.344	63.1 ± 23.6	63.7 ± 21.1	-0.030
Diabetes mellitus						
No	77 (19.6)	83 (25.3)	-0.137	22.2	22.2	0.000
Yes	177 (45.0)	140 (42.7)	0.057	44.0	44.0	0.000
Prediabetes	139 (35.4)	105 (32.0)	0.071	33.8	33.8	0.000
Hypertension	352 (89.6)	304 (92.7)	-0.110	90.5	91.5	-0.031
UTI over previous 12 months	39 (9.9)	31 (9.5)	0.016	9.1	9.3	-0.007
Dyslipidemia	290 (73.8)	246 (75.0)	-0.028	73.7	75.5	-0.042
Atrial fibrillation	202 (51.4)	169 (51.5)	-0.002	50.7	52.7	-0.039
Hemoglobin (g/L)	134 ± 19	134 ± 18	-0.003	134 ± 18	133 ± 18	0.071
Anemic	149 (37.9)	103 (31.4)	0.137	35.9	34.7	0.026
Asthma/COPD	61 (15.5)	40 (12.2)	0.096	14.9	12.9	0.062
Coronary artery disease	190 (49.6)	164 (50.0)	-0.008	48.9	50.3	-0.027
ACS/AMI over 12 months	6 (1.5)	5 (1.5)	0.000	1.5	1.4	0.004
ACS/AMI at start of SGLT2i	14 (3.6)	21 (6.4)	-0.131	4.3	5.3	-0.046
History of CVI/TIA	50 (12.7)	29 (8.8)	0.125	12.0	10.0	0.065
CVI/TIA at start of SGLT2i	2 (0.5)	3 (0.9)	-0.048	0.3	0.9	-0.064
DVT/PE over 12 months	4 (1.0)	11 (3.3)	-0.160	1.1	3.0	0.098
Peripheral artery disease	86 (21.9)	65 (19.8)	0.051	21.1	21.4	-0.003
HHF over 12 months						
None	177 (45.0)	184 (56.1)	-0.222	49.1	51.1	-0.040
One	156 (39.7)	114 (34.8)	0.102	37.8	37.5	0.007
Two	48 (12.2)	26 (7.9)	0.143	10.4	9.7	0.022
Three	10 (2.5)	4 (1.2)	0.098	2.5	1.7	0.057
Four	2 (0.5)	0	0.101	0.2	0	0.048
ER for HF over 12 months						
None	176 (44.8)	170 (51.8)	-0.141	47.6	49.0	-0.028
One	159 (40.5)	130 (39.6)	0.017	39.1	40.9	-0.037
Two	49 (12.5)	27 (8.2)	0.139	11.5	9.7	0.059
Three	6 (1.5)	1 (0.3)	0.129	1.2	0.4	0.089
Four	3 (0.8)	0	0.124	0.5	0	0.088
HHF at start of SGLT2i	117 (29.8)	85 (25.9)	0.086	28.0	28.9	-0.021
CRT or ICD or pacemaker	102 (25.9)	75 (22.9)	0.072	24.5	25.7	-0.028
ACEi or AT1 antagonist	221 (56.2)	200 (61.0)	-0.096	58.0	59.9	-0.038
Sacubitril-valsartan	128 (32.6)	100 (30.5)	0.045	31.1	32.1	-0.021
MRA	321 (81.7)	265 (80.8)	0.023	80.3	82.3	-0.051
Diuretic	326 (82.9)	239 (72.9)	0.245	79.6	77.1	0.061
Beta-blocker	354 (90.1)	301 (91.8)	-0.059	89.9	91.8	-0.067
CCB	80 (20.4)	74 (22.6)	-0.054	21.7	20.6	0.027
Dyslipidemia treatment						
None	128 (32.6)	93 (28.4)	0.092	32.1	29.2	0.065
High-potency statin	243 (61.8)	209 (63.7)	-0.039	61.6	63.0	-0.027
Statin + ezetimib	22 (5.6)	26 (7.9)	-0.093	6.2	7.9	-0.067
Metformin	83 (21.1)	89 (27.1)	-0.141	22.9	24.9	-0.047
GLP-1 agonist	26 (6.6)	47 (14.3)	-0.254	9.6	10.6	-0.033
Inzulin	26 (6.6)	18 (5.5)	0.057	6.1	5.9	0.009
Other antidiabetic	29 (7.4)	17 (5.2)	0.091	5.9	4.9	0.043
Anticoagulation						
None	176 (44.8)	155 (47.3)	-0.050	45.0	44.9	0.002
Warfarin	54 (13.7)	36 (11.0)	0.084	13.9	12.0	0.058
DOAC	163 (41.5)	137 (41.7)	-0.006	41.1	43.1	-0.040
Dual antiplatelet/equivalent						
None	347 (82.4)	273 (83.2)	-0.021	82.2	83.8	-0.043
Aspirin + P2Y12 antagonist	42 (10.7)	39 (11.9)	-0.038	10.8	11.2	-0.012
OAC + P2Y12 antagonist	27 (6.9)	16 (4.9)	0.085	7.0	5.0	0.085

*Abbreviations: ACEi/AT1 – angiotensin converting enzyme inhibitor/angiotensin receptor type 1; ACS/AMI – acute coronary syndrome/acute myocardial infarction; BMI – body mass index; CCB – calcium channel blocker; COPD – chronic obstructive pulmonary disease; CRT – cardiac resynchronization therapy; CVI/TIA – cerebrovascular insult/transitory ischemic attack; DOAC – direct oral anticoagulants; DVT/PE – deep vein thrombosis/pulmonary embolism; eGFR – estimated glomerular filtration rate; ER – emergency department visit; GLP1 – glucagon-like polypeptide 1; HF p/mr/r EF – heart failure with preserved/moderately reduced /reduced ejection fraction; HHF – hospitalization for heart failure; ICD – implantable cardioverter defibrillator; LVEF – left ventricular ejection fraction; MRA – mineralocorticoid receptor antagonist; NT-proBNP – N-terminal pro-brain natriuretic polypoeptide; NYHA – New York Heart Association; OAC – oral anticoagulant; UTI – urinary tract infection.

†Raw data (before weighting) are count (percent), median (range) or mean±SD (range). Adjusted Dapa (after weighting) data are (weighted) percentages, mean±SD, or geometric mean (for ln-transformed continuous variables). Shown are also standardized mean differences (d): where d <0.1, balance between DAPA and EMPA-treated subjects is considered adequate.

‡Effective sample size after weighting: dapagliflozin n = 360 (weights mean 1.0 SD 0.304, range 0.19-2.00), empagliflozin n = 293 (weights mean 1.0 SD 0.346, range 0.12-2.09).

**Table 2:** Patient characteristics in Study 2 by sodium-glucose transporter 2 inhibitor (SGLT2i) – dapagliflozin (DAPA) or empagliflozin (EMPA) – before and after optimization weighting*

	Before weighting			After weighting		
	DAPA	EMPA	d	DAPA	EMPA	d
N	124	116	—	124	116	—
Age (years)	66 ± 13 (31-87)	64 ± 12 (27-89)	0.112	65 ± 14	65 ± 12	0.029
Male	86 (69.4)	85 (73.3)	-0.087	69.7	71.9	-0.048
HF r EF (LVEF≤40%)	105 (84.7)	67 (57.8)	0.623	71.7	71.6	0.001
HF mr/p EF (LVEF>40%)	19 (15.3)	49 (42.2)	-0.623	28.3	28.4	-0.001
LVEF 50%-78%	12 (9.7)	29 (25.0)	—	18.8	16,1	—
LVEF 41%-49%	7 (5.6)	20 (17.2)	—	9.5	12.3	—
LVEF≤40%	105 (84.7)	67 (57.8)	—	71.7	71.6	—
LVEF (%)	33.4 ± 9.9 (15-62)	40.7 ± 12.7 (15-75)	-0.647	36.8 ± 11.0	37.1 ± 12.0	-0.031
NYHA 1	6 (4.8)	7 (6.0)	-0.053	5.8	4.5	0.059
NYHA 2	42 (33.9)	59 (50.9)	-0.349	41.1	43.1	-0.040
NYHA 3	63 (50.8)	42 (36.2)	0.298	44.3	42.7	0.033
NYHA 4	13 (10.5)	8 (6.9)	0.128	8.8	9.7	-0.035
NT-proBNP (pg/mL)	4174 (34-35000)	2639 (250-35000)	—	3271	3301	—
Ln(NT-proBNP)	8.297 ± 1.228	7.912 ± 1.112	0.329	8.093 ± 1.263	8.102 ± 1.086	-0.008
BMI (kg/m^2^)	28.4 (15-50.4)	27.9 (19.5-55.4)	—	28.7	28.5	—
Ln(BMI)	3.364 ± 0.213	3.341 ± 0.170	0.115	3.356 ± 0.208	3.350 ± 0.172	0.031
Chronic kidney disease	33 (26.6)	34 (29.3)	-0.060	27.9	27.9	0.000
eGFR (mL/min/1.73m^2^)	71.8 ± 22.0	74.0 ± 21.6	-0.099	72.3 ± 23.3	72.9 ± 21.5	-0.029
Diabetes mellitus						
No	37 (29.8)	41 (35.3)	-0.118	32.5	32.5	0.000
Yes	41 (33.1)	48 (41.4)	-0.173	37.1	37.1	0.000
Prediabetes	46 (37.1)	27 (23.3)	0.304	30.4	30.4	0.000
Hypertension	89 (71.8)	91 (78.5)	-0.155	74.0	76.0	-0.047
Dyslipidemia	69 (55.7)	38 (30.7)	0.355	62.2	65.2	-0.064
Atrial fibrillation	84 (72.4)	27 (23.3)	1.100	26.1	27.2	-0.025
Hemoglobin (g/L)	137 ± 18	139 ± 19	-0.133	137 ± 18	138 ± 20	-0.051
Anemic	37 (29.8)	36 (31.0)	-0.026	29.4	31.4	-0.043
Coronary artery disease	61 (49.2)	79 (68.1)	-0.391	57.5	59.9	-0.048
ACS/AMI over 12 months	2 (1.6)	0	—	1.2	0	—
ACS/AMI at start of SGLT2i	8 (6.5)	17 (14.7)	—	9.1	11.6	—
Carotide/peripheral artery	25 (20.2)	16 (13.8)	0.170	18,1	16.1	0.054
History of CVI/TIA	14 (11.3)	2 (1.7)	—	9.2	2.4	—
CVI/TIA at start of SGLT2i	1 (0.8)	0	—	0.5	0	—
Peripheral artery disease	15 (12.1)	15 (12.9)	—	12.5	14.2	—
HHF at start of SGLT2i	71 (57.3)	39 (33.6)	0.488	46.8	44.8	0.042
CRT or ICD or pacemaker	15 (12.1)	14 (12.1)	0.000	11.6	12.6	-0.030
UTI over previous 12 mo	11 (8.9)	9 (7.8)	0.040	8.5	9.1	-0.021
ACEi or AT1 antagonist	87 (70.2)	91 (78.5)	-0.191	73.2	75.0	-0.042
Sacubitril-valsartan	29 (23.4)	17 (14.7)	0.224	18.8	20.2	-0.035
MRA	111 (89.5)	86 (74.1)	0.407	82.5	81.0	0.038
Diuretic	96 (77.4)	65 (56.0)	0.466	67.3	68.5	-0.026
Beta-blocker	117 (94.4)	102 (87.9)	0.228	92.5	90.0	0.087
CCB	25 (20.2)	21 (18.1)	0.050	20.1	18.2	0.049
Dyslipidemia treatment	83 (66.9)	88 (75.9)	-0.199	72.0	69.8	0.049
None	41 (33.1)	28 (24.1)	—	28.0	30.2	—
High-potency statin	76 (61.3)	83 (71.6)	—	63.6	66.6	—
Statin + ezetimib	7 (5.7)	5 (4.3)	—	8.4	3.2	—
Antidiabetic treatment	24 (19.4)	37 (31.9)	-0.290	24.4	26.5	-0.048
Metformin	16 (12.9)	32 (27.6)	—	14.2	22.2	—
GLP-1 agonist	11 (8.9)	8 (6.9)	—	12.2	6.3	—
Inzulin	4 (3.2)	6 (5.2)	—	4.4	4.8	—
Other	4 (3.2)	6 (5.2)	—	4.2	3.8	—
Anticoagulation	46 (37.1)	31 (26.7)	0.224	33.1	31.1	0.044
None	78 (62.9)	85 (73.3)	—	66.9	68.9	—
Warfarin	6 (4.8)	6 (5.2)	—	5.9	6.2	—
DOAC	40 (32.3)	25 (21.5)	—	27.2	24.8	—
Antiplatelet	40 (32.3)	65 (56.0)	-0.493	42.2	45.2	-0.063
None	84 (67.7)	51 (44.0)	—	57.8	54.8	—
Aspirin + P2Y12 antagonist	32 (25.8)	54 (46.5)	—	36.3	36.0	—
P2Y12 antagonist	8 (6.5)	11 (9.5)	—	5.9	9.2	—

*Abbreviations: ACEi/AT1 – angiotensin converting enzyme inhibitor/angiotensin receptor type 1; ACS/AMI – acute coronary syndrome/acute myocardial infarction; BMI – body mass index; CCB – calcium channel blocker; COPD – chronic obstructive pulmonary disease; CRT – cardiac resynchronization therapy; CVI/TIA – cerebrovascular insult/transitory ischemic attack; DOAC – direct oral anticoagulants; DVT/PE – deep vein thrombosis/pulmonary embolism; eGFR – estimated glomerular filtration rate; ER – emergency department visit; GLP1 – glucagon-like polypeptide 1; HF p/mr/r EF – heart failure with preserved/moderately reduced /reduced ejection fraction; HHF – hospitalization for heart failure; ICD – implantable cardioverter defibrillator; LVEF – left ventricular ejection fraction; MRA – mineralocorticoid receptor antagonist; NT-proBNP – N-terminal pro-brain natriuretic polypeptide; NYHA – New York Heart Association; OAC – oral anticoagulant; UTI – urinary tract infection

†Raw data (before weighting) are count (percent), median (range) or mean±SD (range). Adjusted (after weighting) data are (weighted) percentages, mean±SD, or geometric mean (for ln-transformed variables). Shown are also standardized mean differences (d): where d <0.1, balance between DAPA and EMPA-treated subjects is considered adequate. Considering the limited number of patients, some characteristics were collapsed into coarser categories for weighting (denoted by the depicted d-values), but data on included subcagetories (not used for weighting) are also shown.

‡Effective sample size after weighting: dapagliflozin n = 96.6 (weights mean 1.0 SD 0.537, range 0.13-2.82), empagliflozin n = 93.1 (weights mean 1.0 SD 0.498, range 0.10-2.15).

### Primary and secondary outcomes: primary analysis

**Figure 2:**
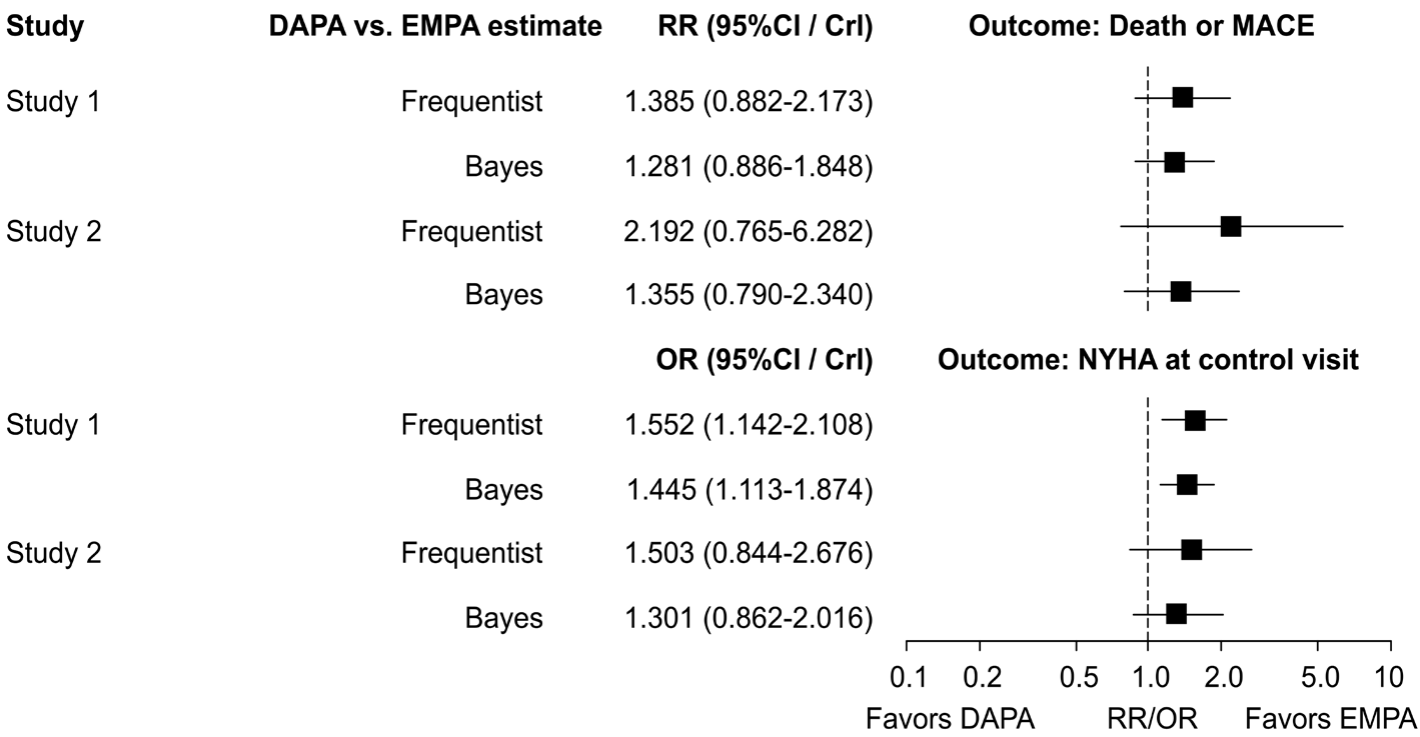
Summary of the adjusted (weighted) primary analysis (each study separately) of the primary (all-cause mortality/major adverse cardiovascular events, MACE) and secondary (New York Heart Association, NYHA, class at the control visit) outcomes. Effect estimates are for the dapagliflozin (DAPA) vs empagliflozin (EMPA) contrast: relative risks (RR) for the primary and odds ratios (OR) for the secondary outcome (OR>1.0 indicates higher odds of a worse NYHA class). Bayesian credible intervals are the highest posterior density intervals.

Control visits were performed within a narrow range of 161/163 to 196/198 days since the start of SGLT2i treatment (Table 3). In Study 1, 82 patients (11.4%) experienced the primary outcome: 38 patients died and 53 experienced at least one MACE event, predominantly HF deterioration (Table 3). The incidence of the primary outcome and its components was somewhat higher in dapagliflozin-prescribed than in empagliflozin -prescribed patients (13.7% vs 8.5% raw data, 12.8% vs 9.2% weighted data) (Table 3). Also, dapagliflozin-prescribed patients were more likely to present with a worse NYHA class (Table 3). In Study 2, only 20 patients experienced the primary outcome (8 died, 14 with at least one MACE event) (Table 3) – still, the incidence of the primary outcome was higher in dapagliflozin-prescribed patients (11.3% vs 5.2% regarding both raw and weighted data) (Table 3). Again, dapagliflozin-prescribed patients were more likely to present with a worse NYHA class (Table 3). For the primary outcome, adjusted relative risk point-estimates (dapagliflozin vs empagliflozin) were all >1.28 in both studies (Figure 2), but with wide confidence intervals (limited sample size/number of events). For the secondary outcome, in Study 1 both the adjusted frequentist (OR = 1.552, 95%CI 1.142-2.108) and the Bayesian estimates (OR = 1.445, 95%CrI 1.113-1.874) suggested higher odds of a worse NYHA class in dapagliflozin-prescribed patients (Figure 2). Point-estimates were similar in Study 2, but confidence intervals extended to <1.0 (Figure 2).

**Table 3:** Primary and secondary outcomes in Study 1 and Study 2: cumulative incidence of death/major adverse cardiovascular events (MACE) between start of treatment with dapagliflozin (DAPA) or empagliflozin (EMPA) and control visit (at 6 months ±2 weeks) (primary), and New York Heart Association (NYHA) class at the control visit (secondary). Data are count (percent) or weighted percent, and median (range) for timing of the control visit

	Study 1				Study 2			
	Raw data (unadjusted)		Weighted data (adjusted)		Raw data (unadjusted)		Weighted data (adjusted	
	DAPA	EMPA	DAPA	EMPA	DAPA	EMPA	DAPA	EMPA
N	393	328	393	328	124	116	124	116
Control visit timing (days)	184 (163-196)	184 (161-196)	184 (163-196)	184 (161-196)	184 (163-197)	183 (165-198)	184 (163-197)	183 (165-198)
Died or MACE to day 160	54 (13.7)	28 (8.5)	12.8%	9.2%	14 (11.3)	6 (5.2%)	11.3%	5.2%
Died	25 (6.4)	13 (4.0)	5.9%	4.4%	6 (4.9)	2 (1.7%)	3.7%	2.2%
MACE	36 (9.2)	17 (5.2)	8.3%	5.6%	10 (8.1)	4 (3.5%)	9.1%	2.9%
Heart failure deterioration	27	9	—	—	4	3	—	—
Acute myocardial infarction	4	3	—	—	4	0	—	—
Cerebrovascular insult	5	4	—	—	2	1	—	—
Deep vein thrombosis	0	1	—	—	—	—	—	—
NYHA class at control visit								
1	123 (31.3)	153 (46.6)	34.7%	43.9%	63 (51.2)	78 (69.0)	57.0%	65.1%
2	190 (48.4)	137 (41.8)	46.9%	42.3%	47 (38.2)	31 (27.4)	33.1%	30.5%
3	43 (10.9)	22 (6.7)	9.4%	8.5%	7 (5.7)	1 (0.9)	6.1%	1.7%
4	12 (3.0)	3 (0.9)	3.1%	0.9%	0	1 (0.9)	0	0.5%
Died (considered as class ‘5′)	25 (6.4)	13 (4.0)	5.9%	4.4%	6 (4.9)	2 (1.7)	3.7%	2.2%

### Primary and secondary outcomes: secondary analysis and sensitivity to unmeasured confounding

**Figure 3:**
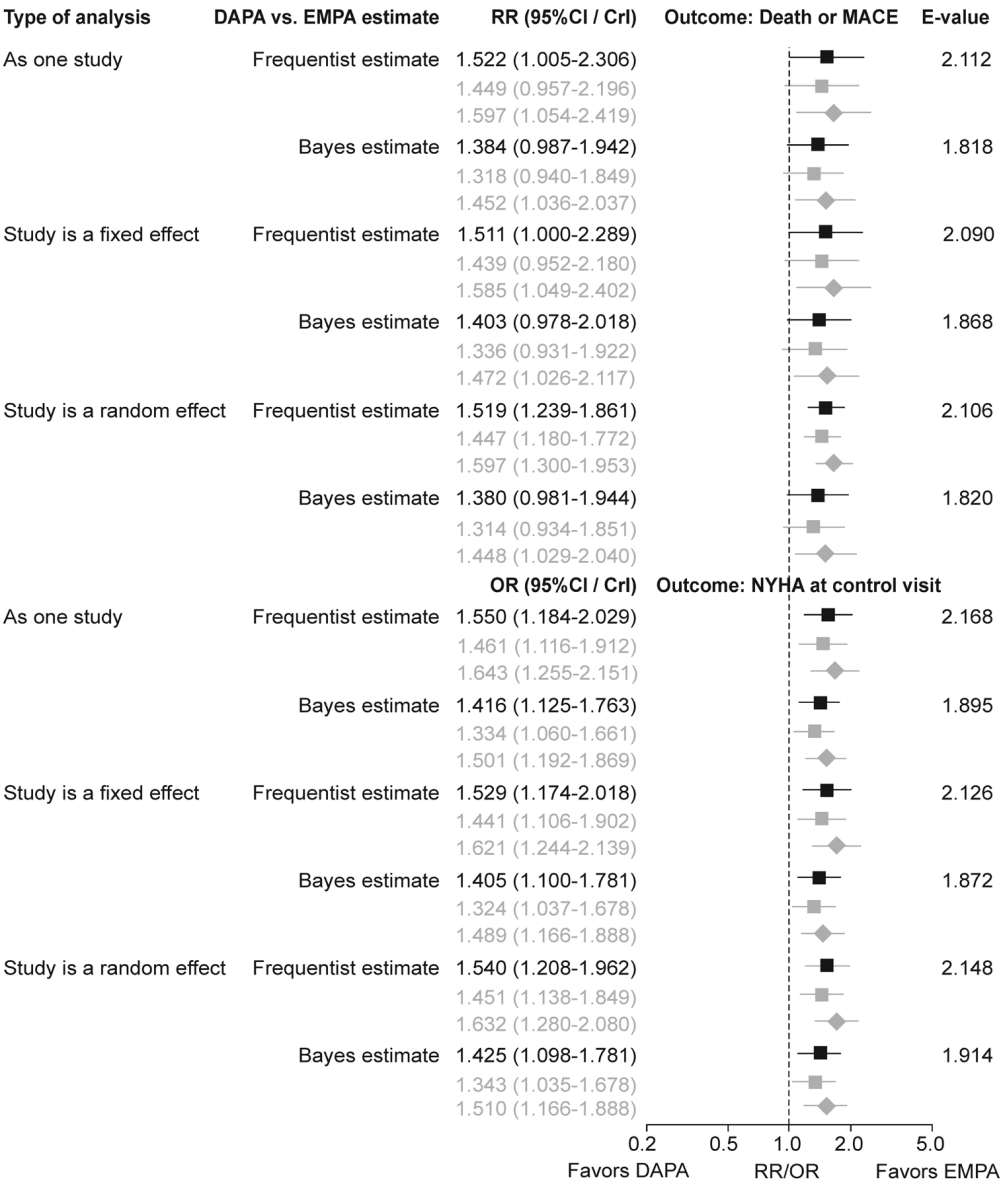
Summary of the adjusted (weighted) secondary analysis (pooled data) of the primary (all-cause mortality/major adverse cardiovascular events, MACE) and secondary (New York Heart Association, NYHA, class at the control visit) outcomes with sensitivity to unmeasured confounding. Effect estimates are for the dapagliflozin (DAPA) vs empagliflozin (EMPA) contrast: relative risks (RR) for the primary and odds ratios (OR) for the secondary outcome (OR>1.0 indicates higher odds of a worse NYHA class). Bayesian credible intervals are the highest posterior density intervals. Black squares (bars) denote observed effects. Gray squares (bars) and font denote estimates corrected for a hypothetical bias disfavoring dapagliflozin: (i) we assumed a strong residual confounding bias with an effect of RR = 1.40 for the primary outcome and OR = 1.50 for the secondary outcome, where the overall prevalence of the “biasing variables” is 35%, with a higher prevalence in dapagliflozin-treated than in empagliflozin-treated patients (ratio 1.5:1.0), ie, dapagliflozin 41.4% vs empagliflozin 27.5%. The observed effects were corrected for this hypothetical bias. Gray diamonds (bars) and font denote estimates corrected for the same hypothetical bias but under the assumption that it disfavored empagliflozin: “biasing variables” were assumed more prevalent in empagliflozin vs dapagliflozin-treated patients (ratio 1.5:1.0), ie, empagliflozin 42.3% vs dapagliflozin 28.6%. The E-values denote (on a risk ratio scale) the size of a confounding effect needed to explain away the largest part of the observed point-estimates, ie, to “push” them to RR/OR = 1.10.

In the combined weighted Study 1 and Study 2 data, all considered covariates were adequately balanced (all d <0.1) between dapagliflozin-prescribed (n = 517) and empagliflozin-prescribed (n = 444) patients (Table 4). Regardless of whether analyzed as a “single study,” or as a fixed-effect or mixed-effects one-stage individual patient data meta-analysis, regarding the primary outcome, all frequentist estimates were entirely ≥1.0, suggesting around 51% higher risk of death/MACE in dapagliflozin-prescribed patients (Figure 3). Bayesian estimates were somewhat lower, with lower limits of the 95%CrIs falling slightly <1.0 (Figure 3). Regarding the secondary outcome, all frequentist and Bayesian estimates were entirely >1.0, suggesting around 54% (frequentist) or around 42% (Bayes) higher odds of having worse NYHA class in the dapagliflozin-prescribed patients (Figure 3). Estimates corrected for a hypothetical strong confounding bias “disfavoring” dapagliflozin were only slightly smaller than the observed ones, and remained mainly entirely >1.0 or with lower bounds of the 95%CI/CrI only slightly <1.0 (Figure 3, gray squares). Estimates corrected for the same hypothetical bias but “disfavoring” empagliflozin were slightly greater than the observed ones (Figure 3, gray diamonds).The E-values suggested that a very strong confounding bias (association with both dapagliflozin and poor outcomes, on the relative risk scale between 1.820 and 2.168) would be needed to explain away the largest part of the observed point-estimates, ie, to “push” the point-estimates to RR/OR = 1.10 (Figure 3).

**Table 4:** Patient characteristics – combined weighted Study 1 and Study 2 data by dapagliflozin (DAPA) or empagliflozin (EMPA). Data are (weighted) percentages, mean±SD, or geometric mean (for ln-transformed variables). Shown are also standardized mean differences (d): where d <0.1, balance between DAPA- and EMPA-treated patients is considered adequate*

	DAPA	EMPA	d
N	517	444	—
Age (years)	68 ± 12	68 ± 12	0.005
SGLT2i start lagged vs other treatment (Study 1)	76.0	73.9	0.049
SGLT2i start with other treatments (Study 2)	24.0	26.1	-0.049
Male	66.6	68.7	0.045
HF r EF (LVEF≤40%)	61.1	61.4	-0.004
HF mr/p EF (LVEF>40%)	38.9	38.6	0.004
LVEF 50%-78%	25.1	24.2	0.020
LVEF 41%-49%	13.8	14.5	-0.017
LVEF≤40%	61.1	61.3	-0.006
LVEF (%)	39.4 ± 12.4	39.7 ± 11.8	-0.024
NYHA 1	6.3	4.7	0.067
NYHA 2	47.8	48.9	-0.021
NYHA 3	40.1	39.9	0.003
NYHA 4	5.8	6.5	-0.026
NT-proBNP (pg/mL)	2450	2545	—
Ln(NT-proBNP)	7.804 ± 1.291	7.842 ± 1.193	-0.031
BMI (kg/m^2^)	29.1	29.2	—
Ln(BMI)	3.371 ± 0.188	3.373 ± 0.184	-0.011
Chronic kidney disease	39.1	38.7	0.007
eGFR (mL/min/1.73m^2^)	65.3 ± 23.8	66.1 ± 21.5	-0.038
Diabetes mellitus			
No	24.7	24.9	-0.005
Yes	42.3	42.2	0.006
Prediabetes	33.0	32.9	0.002
Hypertension	86.5	87.4	-0.027
Dyslipidemia	71.0	72.9	-0.042
Atrial fibrillation	44.8	46.1	-0.024
Hemoglobin (g/L)	135 ± 18.4	135 ± 18.7	0.030
Anemic	34.4	33.9	0.011
Coronary artery disease	52.0	53.4	-0.028
ACS/AMI over 12 months	1.4	1.1	0.031
ACS/AMI at start of SGLT2i	5.5	6.9	-0.060
Carotide/peripheral artery disease	25.6	24.7	0.020
History of CVI/TIA	11.3	8.0	0.098
CVI/TIA at start of SGLT2i	0.4	0.6	-0.034
Peripheral artery disease	19.1	19.5	-0.009
COPD /asthma	13.7	11.0	0.084
HHF / 12 months (includes index HHF for Study 2)			
0	55.5	50.6	0.042
1	33.7	40.7	-0.060
2	9.3	7.4	0.037
3	1.1	1.3	-0.053
4	0.4	0	0.042
ER HF/ 12 months (includes index visit for Study 2)			
0	55.5	57.7	-0.044
1	33.7	34.7	-0.022
2	9.3	7.3	0.074
3	1.1	0.3	0.085
4	0.4	0	0.077
HHF at start of SGLT2i	32.5	33.1	-0.012
CRT or ICD or pacemaker	21.4	22.3	-0.021
UTI over previous 12 months	9.0	9.3	-0.010
ACEi or AT1 antagonist	61.7	63.8	-0.045
Sacubitril-valsartan	28.2	29.0	-0.018
MRA	80.8	81.9	-0.025
Diuretic	76.7	74.9	0.042
Beta-blocker	90.5	91.3	-0.029
CCB	21.4	20.0	0.034
Dyslipidemia treatment	68.8	70.6	-0.038
None	31.2	29.4	0.038
High-potency statin	62.1	63.9	-0.037
Statin + ezetimib	6.7	6.7	0.000
Antidiabetic treatment	31.7	30.5	0.026
Metformin	20.8	24.1	-0.080
GLP-1 agonist	10.3	9.5	0.025
Insulin	5.7	5.6	0.004
Other	5.5	4.6	0.039
Anticoagulation	49.7	48.8	0.019
None	50.3	51.2	-0.019
Warfarin	12.0	10.5	0.049
DOAC	37.7	38.3	-0.012
Antiplatelet/equivalent	23.7	23.8	-0.003
None	76.3	76.2	0.003
Aspirin + P2Y12 antagonist	17.0	17.7	-0.020
OAC + P2Y12 antagonist or P2Y12 antagonist	6.7	6.1	0.025

*Abbreviations: ACEi/AT1 – angiotensin converting enzyme inhibitor/angiotensin receptor type 1; ACS/AMI – acute coronary syndrome/acute myocardial infarction; BMI – body mass index; CCB – calcium channel blocker; COPD – chronic obstructive pulmonary disease; CRT – cardiac resynchronization therapy; CVI/TIA – cerebrovascular insult/transitory ischemic attack; DOAC – direct oral anticoagulants; DVT/PE – deep vein thrombosis/pulmonary embolism; eGFR – estimated glomerular filtration rate; ER – emergency department visit; GLP1 – glucagon-like polypeptide 1; HF p/mr/r EF – heart failure with preserved/moderately reduced /reduced ejection fraction; HHF – hospitalization for heart failure; ICD – implantable cardioverter defibrillator; LVEF – left ventricular ejection fraction; MRA – mineralocorticoid receptor antagonist; NT-proBNP – N-terminal pro-brain natriuretic polypeptide; NYHA – New York Heart Association; OAC – oral anticoagulant; UTI – urinary tract infection

### Other outcomes

NT-proBNP levels, LVEF, and eGFR values at the control visit (survivors only) were similar between dapagliflozin-prescribed and empagliflozin -prescribed patients in both studies (Supplemental Material 1(Supplemental Material 1)).

### Exploration of effect modification by chronic kidney disease and ejection fraction

**Figure 4:**
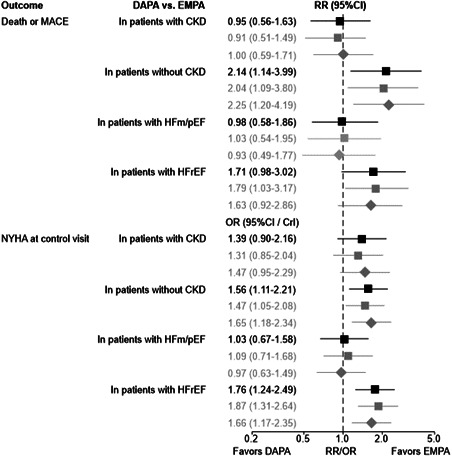
Summary of the analysis of chronic kidney disease (CKD), and of left ventricular ejection fraction (mildly reduced/preserved [HFm/pEF] or reduced [HFrEF]) as moderators of the dapagliflozin (DAPA) vs empagliflozin (EMPA) differences in the primary (incidence of all-cause mortality/major adverse cardiovascular events, MACE) and secondary (New York Heart Association, NYHA, class at the control visit) outcomes. Effect estimates are for the DAPA vs EMPA contrast: relative risks (RR) for the primary and odds ratios (OR) for the secondary outcomes (pooled data on prevalent and incident patients). Black squares (bars) denote observed effects. Gray squares and diamonds (bars) and font denote estimates corrected for a hypothetical bias disfavoring dapagliflozin or empagliflozin, respectively (see footnote to Figure 3 for details).

Fewer patients had CKD (dapagliflozin n = 232, empagliflozin n = 142) or HFm/pEF (dapagliflozin n = 158, empagliflozin n = 215) than a preserved renal function (dapagliflozin n = 285, empagliflozin n = 302) or HFrEF (dapagliflozin 359, empagliflozin n = 229). Across the subsets, dapagliflozin and empagliflozin patients mildly to moderately differed on a range of baseline covariates, but were adequately balanced on all of them (Supplemental Material 2(Supplemental Material 2), Table B1 – Table B4). In patients with CKD and in those with HFm/pEF, raw data indicated somewhat higher incidence of the primary outcome and of worse NYHA class in dapagliflozin- vs empagliflozin-prescribed patients (Table 5), but weighted data did not (Table 5). In patients with a preserved renal function, and in those with HFpEF, both raw and weighted data indicated worse outcomes with dapagliflozin vs empagliflozin (Table 5). In the fully adjusted analysis (Figure 4): i) the primary outcome was similar for the two treatments in patients with CKD (RR = 0.95, 95%CI 0.56-1.63) and in those with HFm/pEF (RR = 0.98, 0.58-1.86), but was markedly higher with dapagliflozin in patients without CKD (RR = 2.14, 1.14-3.99) and in those with HFrEF (RR = 1.71, 0.98-3.02); ii) in both patients with (OR = 1.39, 0.90-2.16) and without CKD (1.56, 1.11-2.21), dapagliflozin-prescribed patients had higher odds of a worse NYHA class than empagliflozin-prescribed patients. In patients with HFm/pEF, there was no difference between the treatments (OR = 1.03, 0.67-1.58), but in patients with HFrEF, the odds of a worse NYHA class were higher with dapagliflozin than with empagliflozin (OR = 1.76, 1.24-2.49).

**Table 5:** Primary and secondary outcomes in patients with chronic kidney disease (CKD), patients with a preserved renal function, patients with mildly reduced or preserved left ventricular ejection fraction (LVEF) (HFm/pEF), and patients with a reduced ejection fraction (HFrEF): cumulative incidence of death/major adverse cardiovascular events (MACE) between the start of treatment with dapagliflozin (DAPA) or empagliflozin (EMPA) and control visit (at 6 months ±2 weeks) (primary), and New York Heart Association (NYHA) class at the control visit (secondary). Data are count (percent) or weighted percent

Subsets of CKD	Patients with CKD				Patients with preserved renal function			
	Raw data		Weighted data		Raw data		Weighted data	
	DAPA	EMPA	DAPA	EMPA	DAPA	EMPA	DAPA	EMPA
N	232	142	232	142	285	302	285	302
Died or MACE	35 (15.1)	19 (13.4)	14.2%	14.9%	33 (11.6)	15 (5.0)	11.0%	5.1%
Died	17 (7.3)	9 (6.4)	7.0%	7.6%	14 (4.9)	6 (2.0)	4.0%	2.1%
MACE	23 (9.1)	12 (8.4)	9.2%	8.7%	23 (8.1)	10 (3.3)	8.1%	3.4%
NYHA class								
1	53 (22.8)	50 (35.5)	24.9%	35.7%	133 (46.7)	181 (59.9)	48.4%	56.9%
2	128 (55.2)	68 (48.2)	54.6%	45.7%	109 (38.2)	101 (33.4)	38.4%	36.0%
3	27 (11.6)	12 (8.5)	10.7%	10.0%	24 (8.4)	12 (4.0)	7.5%	4.3%
4	7 (3.0)	2 (1.4)	2.9%	1.0%	5 (1.7)	2 (0.7)	1.7%	0.7%
Died (class ‘5′)	17 (7.3)	9 (6.4)	7.0%	7.6%	14 (4.9)	6 (2.0)	4.0%	2.1%
Subsets of LVEF	Patients with HFm/pEF				Patients with HFrEF			
	Raw data		Weighted data		Raw data		Weighted data	
	DAPA	EMPA	DAPA	EMPA	DAPA	EMPA	DAPA	EMPA
N	158	215	158	215	359	229	359	229
Died or MACE	20 (12.7)	19 (8.8)	10.9%	11.1%	48 (13.4)	15 (6.6)	13.1%	7.7%
Died	10 (6.3)	7 (3.3)	3.9%	4.0%	21 (5.9)	8 (3.5)	5.7%	4.3%
MACE	11 (7.0)	15 (7.0)	7.3%	8.7%	35 (9.8)	7 (3.1)	9.6%	3.4%
NYHA class								
1	65 (41.1)	115 (53.5)	47.2%	48.2%	121 (33.7)	116 (50.9)	35.4%	48.3%
2	69 (43.7)	79 (36.7)	40.3%	39.4%	168 (46.8)	90 (39.5)	45.9%	39.8%
3	10 (6.3)	11 (5.1)	6.7%	7.0%	41 (11.4)	13 (5.7)	10.6%	7.2%
4	4 (2.5)	3 (1.4)	1.9%	1.5%	8 (2.2)	1 (0.4)	2.4%	0.4%
Died (class ‘5′)	10 (6.3)	7 (3.3)	3.9%	4.0%	21 (5.9)	8 (3.5)	5.7%	4.3%

## Discussion

The present analysis suggests that patients prescribed with dapagliflozin had a higher risk of a composite of all-cause mortality/incidence or MACE (dominated by HF deterioration) over the initial 6 months of treatment than those prescribed with empagliflozin, and that they were also more likely to present with a worse NYHA class after 6 months of treatment (on the intent-to-treat basis). The estimates were consistent in the two patient subsets (incident and prevalent), justifying pooled analysis to improve precision. Exploratory analysis suggested effect modification: differences between treatments were apparent in patients with preserved renal function (corresponds to eGFR>60 mL/min/1.73 m^2^), and in those with HFrEF but not in patients with CKD or in patients with HFm/pEF. However, patient subsets were limited in size (particularly, the number of patients with CKD and those with HFm/pEF was limited).

Since contrary to our expectations, the observed estimates prompted us to first consider potential biases. Theoretically (single center, limited sample), the observed findings could have been due to chance. Several elements, however, support a reasonable external validity of the study: i) the general, (co)morbidity and treatment (other than SGLT2i) characteristics of the included patients were in agreement with those reported in pivotal trials of dapagliflozin and empagliflozin in this setting (12), and in a recent pharmacoepidemiological study (15); (ii) the primary outcome included all-cause death and MACE, rather than specifically cardiovascular death and hospitalization for HF/or HF-related events typically used in clinical trials in this setting. However, MACE was predominated by “HF worsening events.” Also, all-cause mortality in a real-life setting is likely a more suitable outcome than the cause-specific mortality, since a detailed adjudication is commonly not possible – it is less susceptible to misclassification bias and has a direct clinical/practical meaning. It is therefore justified to consider that the primary outcome in the present analysis captures the same trait as does the one used in randomized trials, and it is unlikely that its definition biased the direction of the estimates. The observed overall raw incidence of 10.6% (102 events/961 patients) over 6 months is in line with plausible expectations, considering 6-month occurrence of “primary composites” in a mixed population of HFpEF, HFmrEF, and HFrEF patients in clinical trials (4-7); iii) the weighted incidence of the primary outcome in the pooled data (12.5% dapagliflozin vs 8.2% empagliflozin) corresponds to 64 vs 36 events – it is unlikely that a few events less or more (by chance) occurring in dapagliflozin or empagliflozin-prescribed patients would have substantially changed the direction of the estimates; iv) the NYHA class outcomes were in the same direction as the primary outcome. We therefore deem it reasonable to view the present data as plausible and reasonably robust, at least considering the entire sample of patients.

We believe that the present analysis was reasonably well protected against major biases imminent to observational data (27): i) differential misclassification of outcomes or confounders was unlikely, since all assessments were done in real-time by experienced cardiologists based on objective standard criteria, and irrespective of the present analysis; ii) the only patient selection criteria were a relatively shorter pre-SGLT2i history of heart failure (in prevalent patients), and the absence of some comorbidities typically also excluded in randomized trials. The definition of the NYHA outcome and the intent-to-treat concept minimized the risk of post-baseline selection; iii) in all analyses, dapagliflozin- and empagliflozin -prescribed patients were closely balanced on a wide range of possible confounders. Still, we assumed the existence of residual confounding, potentially arising from a “cumulative effect” of several factors: cumulative minor differences in some of the balanced confounders, differences in dosing/compliance with treatments other than SGLT2i, and other hypothetical sources of confounding. The bias-corrected estimates only mildly differed from the observed ones, and still pointed to poorer outcomes with dapagliflozin. The E-values indicated that whatever the source of residual confounding might have been, its effect would need to be considerable in order to reduce the point-estimates to 1.10. Overall, while the size of the reported estimates might be debatable, their direction seems to be valid. In this respect, the present observations agree with a recent study (15) based on administrative data that indicated lower risk (by around 10%) of hospitalizations for HF (HHF) and of all-cause death over 12 months in empagliflozin- vs dapagliflozin-prescribed patients. While the present and the reported estimates (15) should not be quantitatively compared due to methodological differences, they both point to the same direction.

In their pivotal placebo-controlled trials, the two SGLT2i displayed apparently similar reductions of the risk of HHF/cardiovascular mortality both in patients with HFrEF and in patients with HFm/pEF (4-7). The pharmacoepidemiological study by Modzelewski et al (15) suggested (similar) superiority of empagliflozin vs dapagliflozin in patients with HRrEF, patients with HFm/pEF, and overall (66% with HFrEF). Generally, discrepancies between estimates from quality randomized trials and non-randomized studies can be commonly explained by systematic errors in the latter (28,29), but no such marked biases appear obvious in the reported observational data (15). We argue that the same applies to the present analysis in the entire patient sample and in patients with HFrEF (60% of all). The presently reported lack of obvious differences between dapagliflozin and empagliflozin in patients with HFm/pEF might not be biased, and agrees with the general messages of the randomized trials, but it is difficult to interpret owing to a very limited number of patients in this subset. The same reasoning is applicable to the observed differences between dapagliflozin and empagliflozin in patients with preserved renal function (60% of all), but not in those with CKD. In the pivotal trials, both in HFrEF and in HFm/pEF (4-7), the benefits of the two SGLT2i (vs placebo) were similar in patients with eGFR≤60 mL/min/1.73 m^2^ and in patients with preserved renal function. In the study by Modzelewski et al (15), renal function was not evaluated as a possible effect modifier, but around 70%-75% of the analyzed patients had reasonably preserved renal function (15). In this context, the present data suggest relative efficacy of the two SGLT2i in patients with CHF conditional on the renal function as a topic that deserves to be investigated.

Empagliflozin shows a greater selectivity to SGLT2 vs SGLT1 (the latter being expressed also in the heart) than dapagliflozin, and it is tempting to assume that different gliflozins might differ in some “mechanistic” details to the extent that could result in clinically relevant differences (8,9), but at present no such prominent property has been identified. Indirect comparisons generated in network meta-analyses of randomized trials of different gliflozins in different clinical settings (diabetes, CKD, CHF) provide circumstantial evidence to support the hypothesis that cardiovascular outcomes might somewhat differ between different compounds (30-32). The present and the published (15) observational direct comparisons of dapagliflozin to empagliflozin support the hypothesis that the efficacy of the two specifically in CHF might differ to a practically relevant extent – a possibility that deserves a proper evaluation.

### Conclusion

In the present single-center registry analysis, incident or prevalent CHF patients prescribed with dapagliflozin were more likely to experience a composite of all-cause death or MACE over the initial 6 months of treatment than their empagliflozin -prescribed peers, and they were also more likely to display a worse NYHA class at 6 months. While the analysis is limited by a relatively limited single-center patient sample and limited follow-up, it is reasonable to consider it internally valid, and the reported estimates agree with recently reported superiority of empagliflozin vs dapagliflozin in treatment of CHF in a large pharmacoepidemiological study based on claims data (15). Taken together, the present and the published data (15) strongly emphasize a need for a direct randomized comparison of empagliflozin and dapagliflozin in CHF patients.
