# Supplementary material for: Dapagliflozin vs empagliflozin in patients with chronic heart failure: a registry analysis
Source: Croat Med J. 2025 Apr;66(2):135–52. doi: 10.3325/cmj.2025.66.135 (PMC12093124; doi:10.3325/cmj.2025.66.135)
Supplement: Supplemental Material 1 [file CroatMedJ_66_s016.pdf]

Supplemental Table 1. Other outcomes in Study 1 and Study 2: raw and adjusted (weighted) data assessed at the control visit, only for survivors. Data are mean±SD, and geometric mean for NT-proBNP.

	Study 1				Study 2			
	Raw data		Weighted (adjusted) data		Raw data		Weighted (adjusted) data	
	DAPA	EMPA	DAPA	EMPA	DAPA	EMPA	DAPA	EMPA
N (survivors)	368	315	368	315	118	114	118	114
NT-proBNP (pg/mL)	1270	916	1119	1060	812.4	621.4	736.6	673.2
Ln(NT-proBNP)	7.148±1.271	6.820±1.279	7.020±1.248	6.966±1.305	6.700±1.382	6.432±1.311	6.602±1.362	6.512±1.293
LVEF (%)	41.6±11.7	45.3±11.2	43.0±11.9	43.6±11.1	41.2±10.7	46.3±11.2	43.7±11.4	44.3±11.1
eGFR (mL/min/1.73 m^2)	60.0±23.2	66.4±21.9	62.7±23.0	63.2±22.5	70.0±23.4	73.5±23.4	70.6±23.7	72.4±22.2

DAPA – dapagliflozin; eGFR – estimated glomerular filtration rate; EMPA – empagliflozin; LVEF – left ventricular ejection fraction; NT-proBNP – N-terminal fragment of the brain natriuretic peptide
