# Supplementary material for: Dapagliflozin vs empagliflozin in patients with chronic heart failure: a registry analysis
Source: Croat Med J. 2025 Apr;66(2):135–52. doi: 10.3325/cmj.2025.66.135 (PMC12093124; doi:10.3325/cmj.2025.66.135)
Supplement: Supplemental Material 2 [file CroatMedJ_66_s017.pdf]

Supplemental Material

Dapagliflozin vs. Empagliflozin in patients with and without chronic kidney disease, and in patients with mildly reduced/preserved (HFm/pEF) and with reduced left ventricular ejection fraction (HFrEF**)**

To explore possible modification of the dapagliflozin vs. empagliflozin outcomes by chronic kidney disease (CKD), and by left ventricular ejection fraction (LVEF), dapagliflozin and empagliflozin-treated patients – both prevalent and incident (i.e., with delayed and early start of SGLT2i treatment) - were mutually balanced on baseline covariates separately in the subset with CKD and in the subset with preserved renal function (no CKD), i.e., in the subset with a preserved ejection fraction (HFm/pEF), and in the subset with a reduced ejection fraction (HFrEF). Since the numbers of patients with CKD and those with HFm/pEF were limited (232 prescribed with dapagliflozin, 142 prescribed with empagliflozin; and 158 prescribed with dapagliflozin, 215 prescribed with empagliflozin, respectively), some of the baseline characteristics were collapsed into coarser categories for weighting. The numbers of patients with a preserved renal function (285 presecribed with dapagliflozin, 302 prescribed with empagliflozin), and those with HFrEF (359 prescribed with dapagliflozin, 229 prescribed with empagliflozin) allowed balancing on all the covariates as in the analyses of the overall data.

Table B1. Characteristics of patients with chronic kidney disease by sodium-glucose transporter 2 inhibitor (SGLT2i) – dapagliflozin (DAPA) or empagliflozin (EMPA) - before and after optimization-based weighting.

Table B2. Characteristics of patients with preserved renal function (no chronic kidney disease) by sodium-glucose transporter 2 inhibitor (SGLT2i) – dapagliflozin (DAPA) or empagliflozin (EMPA) - before and after optimization-based weighting.

Table B3. Characteristics of patients with HFrEF by sodium-glucose transporter 2 inhibitor (SGLT2i) – dapagliflozin (DAPA) or empagliflozin (EMPA) - before and after optimization-based weighting.

Table B4. Characteristics of patients with HFm/pEF by sodium-glucose transporter 2 inhibitor (SGLT2i) – dapagliflozin (DAPA) or empagliflozin (EMPA) - before and after optimization-based weighting.

Table B1. Characteristics of patients with chronic kidney disease, by sodium-glucose transporter 2 inhibitor (SGLT2i) – dapagliflozin (DAPA) or empagliflozin (EMPA) - before and after optimization weighting. Data before weighting are raw data, data after weighting are weighted data: percentages, mean±standard deviation, or geometric mean (% coefficient of variation) for ln-transformed variables. Standardized mean differences (d)<0.1 indicate adequate balance between DAPA and EMPA-treated subjects.

	Before weighting (raw data)			After weighting (weighted data)[1]		
	DAPA	EMPA	d	DAPA	EMPA	d
N	232	142	---	232	142	---
Age (years)	74±9	74±8	0.002	74±9	74±8	0.000
SGLT2i start lagged (Study 1)	85.8	76.1	0.249	82.1	82.1	0.000
SGLT2i start early (Study 2)	14.2	23.9	-0.249	17.9	17.9	0.000
Male	61.2	65.5	-0.089	61.1	65.2	-0.086
HF r EF (LVEF ≤40%)	62.9	56.3	0.135	60.2	60.2	0.000
HF mr/p EF (LVEF >40%)	37.1	43.7	-0.135	39.8	39.8	0.000
LVEF 50-78%	22.8	31.0	-0.184	25.9	25.9	0.000
LVEF 41-49%	14.3	12.7	0.025	13.9	13.9	0.000
LVEF ≤40%	62.9	56.3	0.149	60.2	60.2	0.000
LVEF (%)	38.5±12.4	40.9±12.9	-0.194	39.2±12.7	39.7±12.3	-0.042
NYHA 1	3.9	3.5	0.019	3.7	3.7	0.000
NYHA 2	40.1	44.4	-0.087	41.8	41.8	0.000
NYHA 3	46.5	43.0	0.072	45.1	45.1	0.000
NYHA 4	9.5	9.1	0.011	9.4	9.4	0.000
Ln(NT-proBNP) (pg/mL)	3984 (168)	4100 (188)	-0.025	3984 (183)	4087 (172)	-0.022
Ln(BMI) (kg/m^2)	29.4 (18.4)	29.3 (16.9)	0.013	29.3 (17.1)	29.3 (18.4)	0.000
Diabetes mellitus						
No	15.5	26.8	-0.278	19.9	19.9	0.000
Yes	53.0	43.7	0.188	49.4	49.4	0.000
Prediabetes	31.5	29.5	0.041	30.7	30.7	0.000
Hypertension	93.1	94.4	-0.020	92.6	94.6	-0.020
Dyslipidemia	72.4	76.1	-0.083	72.3	75.3	-0.030
Atrial fibrillation	56.0	61.3	-0.106	58.0	58.0	0.000
Hemoglobin (g/L)	130±19	129±20	0.056	130±19	129±19	0.060
Anemic	44.8	47.2	-0.047	44.0	47.2	-0.066
Coronary artery disease	50.0	52.8	-0.056	50.1	52.1	-0.020
ACS/AMI over 12 months	1.7	0.7	0.093	1.8	0.9	0.078
ACS/AMI at start of SGLT2i	4.3	7.8	-0.145	5.0	6.9	-0.077
Carotide/peripheral artery disease	32.8	29.6	0.056	32.1	30.5	0.035
COPD /asthma	15.5	12.7	0.082	14.2	12.3	0.019
HHF/12 months or at start	63.4	54.2	0.186	61.7	57.3	0.099
ER HF/ 12 months or at start	57.3	52.1	0.105	55.4	53.4	0.020
CRT or ICD or pacemaker	22.4	24.7	-0.053	22.8	23.8	-0.010
ACEi or AT1 antagonist	57.3	62.0	-0.092	58.6	59.7	-0.010
Sacubitril-valsartan	31.9	31.0	0.020	31.1	32.1	-0.010
MRA	77.2	79.6	-0.059	77.6	78.6	-0.010
Diuretic	90.1	84.5	0.168	88.5	87.4	0.010
Beta-blocker	90.1	89.4	0.021	89.9	90.3	-0.005
CCB	22.0	24.6	-0.063	22.5	22.7	-0.002
Dyslipidemia treatment						
None	36.2	31.7	0.095	35.0	33.9	0.010
High-potency statin	59.5	64.8	-0.109	60.7	62.7	-0.020
Statin + ezetimib	4.3	3.5	0.041	4.3	3.4	0.010
Antidiabetic treatment	38.8	35.9	0.060	37.2	38.2	-0.010
Anticoagulation						
None	41.8	33.1	0.181	39.0	38.0	0.021
Warfarin	12.5	13.4	-0.026	12.9	13.0	-0.004
DOAC	45.7	53.5	-0.157	48.1	49.0	-0.018
Antiplatelet/equivalent						
None	80.2	79.6	0.015	79.5	80.4	-0.022
Aspirin + P2Y12 antagonist	12.1	12.0	0.003	11.9	12.4	-0.028
OAC + P2Y12 or P2Y12 t	7.8	8.4	-0.025	8.6	7.2	0.054

[1] Weighting only mildly increased the variance, so that the effective sample sizes were only 5-10% smaller than the actual smple size: for DAPA, effective sample size 219.5, mean weight 1.0, SD 0.239, range 0.44-1.91; for EMPA, effective sample size 123.2, mean weight 1.0, SD 0.388, range 0.10-2.03

ACEi/AT1 – angiotensin converting enzyme inhibitor/angiotensin receptor type 1; ACS/AMI – acute coronary syndrome/acute myocardial infarction; BMI – body mass index; CCB – calcium channel blocker; COPD – chronic obstructive pulmonary disease; CRT – cardiac resynchronization therapy; ER – emergency room visit; HF p/mr/r EF – heart failure with preserved/mildly reduced /reduced ejection fraction; HHF – hospitalization for heart failure; ICD – implantable cardioverter defibrillator; LVEF – left ventricular ejection fraction; MRA – mineralocorticoid receptor antagonist; NT-proBNP – N-terminal pro-brain natriuretic polypeptide; NYHA – New York Heart Association; (D)OAC – (direct) oral anticoagulant

Table B2. Characteristics of patients with preserved renal function (no chronic kidney disease) by sodium-glucose transporter 2 inhibitor (SGLT2i) – dapagliflozin (DAPA) or empagliflozin (EMPA) - before and after optimization weighting. Data before weighting are raw data, data after weighting are weighted data: percentages, mean±standard deviation, or geometric mean (% coefficient of variation) for ln-transformed variables. Standardized mean differences (d)<0.1 indicate adequate balance between DAPA and EMPA-treated subjects.

	Before weighting (raw data)			After weighting (weighted data)[1]		
	DAPA	EMPA	d	DAPA	EMPA	d
N	285	302	---	285	302	---
Age (years)	64±12	65±11	-0.071	65±12	65±12	0.000
SGLT2i start lagged (Study 1)	68.1	72.9	-0.105	70.5	70.5	0.000
SGLT2i start early (Study 2)	31.9	27.1	0.105	29.5	29.5	0.000
Male	71.6	68.9	0.059	69.7	71.2	-0.014
HF r EF (LVEF ≤40%)	74.7	49.7	0.542	61.7	61.7	0.000
HF mr/p EF (LVEF >40%)	25.3	50.3	-0.542	38.3	38.3	0.000
LVEF 50-78%	15.4	31.5	-0.385	23.7	23.7	0.000
LVEF 41-49%	9.8	18.8	-0.260	14.5	14.5	0.000
LVEF ≤40%	74.7	49.7	0.535	61.8	61.8	0.000
LVEF (%)	36.3±11.7	42.8±11.7	-0.556	39.4±12.5	40.0±11.4	-0.050
NYHA 1	6.7	7.3	-0.024	7.0	7.0	0.000
NYHA 2	48.1	57.3	-0.185	52.8	52.8	0.000
NYHA 3	40.0	32.4	0.158	36.1	36.1	0.000
NYHA 4	5.3	3.0	0.115	4.1	4.1	0.000
Ln(NT-proBNP)	2153 (205)	1593 (151)	0.253	1835 (192)	1817 (158)	0.008
Ln(BMI) (kg/m^2)	28.5 (20.1)	29.5 (19.1)	-0.178	28.9 (20.6)	29.1 (18.6)	-0.025
eGFR (mL/min/1.73m^2)	81.0±14.5	80.3±14.5	0.045	80.9±14.6	80.4±14.8	0.030
Diabetes mellitus						
No	27.4	28.5	-0.025	27.9	27.9	0.000
Yes	33.3	41.7	-0.174	37.7	37.7	0.000
Prediabetes	39.3	29.8	0.201	34.4	34.4	0.000
Hypertension	78.9	86.4	-0.199	82.6	84.3	-0.016
Dyslipidemia	67.0	73.5	-0.142	68.9	71.9	-0.030
Atrial fibrillation	38.6	36.1	0.052	37.3	37.3	0.000
Hemoglobin (g/L)	139±17	138±17	0.002	139±17	139±17	0.000
Anemic	28.8	23.8	0.112	27.7	24.7	0.030
Coronary artery disease	50.9	56.6	-0.115	52.5	55.1	-0.026
ACS/AMI over 12 months	1.4	1.3	0.007	1.3	1.3	0.000
ACS/AMI at start of SGLT2i	4.2	8.9	-0.192	4.5	8.1	-0.035
Carotide/peripheral artery disease	22.8	18.9	0.097	22.0	20.4	0.016
History of CVI/TIA	10.5	7.6	0.102	10.3	8.5	0.018
CVI/TIA at start of SGLT2i	0.7	1.0	-0.032	0.4	1.1	-0.007
Peripheral artery disease	15.4	13.6	0.053	16.0	14.0	0.020
COPD /asthma	12.6	8.9	0.119	12.0	9.5	0.025
HHF / 12 months or at start	51.9	38.1	0.2811	46.0	43.5	0.025
0	48.1	61.9	-0.281	54.0	56.5	-0.025
1	41.7	34.4	0.151	37.2	38.8	-0.015
2	8.4	3.3	0.219	7.0	4.5	0.025
3	1.8	0.3	0.141	1.8	0.2	0.016
ER HF/ 12 months or at start	39.6	33.4	0.129	34.9	35.2	-0.016
0	60.3	66.6	-0.129	63.1	64.8	-0.016
1	29.1	29.8	-0.015	28.5	30.1	-0.016
2	9.1	3.3	0.242	7.4	4.9	0.025
3	1.1	0.3	0.087	0.7	0.2	0.005
4	0.4	0	0.084	0.3	0	0.003
HHF at start of SGLT2i	33.3	25.8	0.165	28.9	30.0	-0.011
CRT or ICD or pacemaker	22.8	17.9	0.123	19.8	20.5	-0.007
ACEi or AT1 antagonist	61.4	67.2	-0.122	64.2	64.7	-0.005
Sacubitril-valsartan	29.1	24.2	0.112	25.6	27.6	-0.020

MRA	88.8	78.8	0.273	83.3	84.0	-0.007
Diuretic	74.7	60.9	0.299	68.6	66.6	0.020
Beta-blocker	91.9	91.4	0.019	90.4	92.1	-0.017
CCB	18.9	19.9	-0.023	20.4	18.4	0.020
Dyslipidemia treatment						
None	29.8	25.2	0.104	28.4	27.4	0.010
High-potency statin	63.5	66.2	-0.057	64.4	64.4	0.000
Statin + ezetimib	6.7	8.6	-0.073	7.2	8.2	-0.010
Antidiabetic treatment	22.1	32.4	-0.234	28.4	27.0	0.014
Metformin	15.8	27.8	-0.294	21.0	23.0	-0.020
GLP-1 agonist	4.9	11.9	-0.254	7.5	9.5	-0.020
Insulin	3.9	4.6	-0.039	4.6	4.8	-0.001
Other	2.8	4.6	-0.097	3.1	3.4	-0.004
Anticoagulation						
None	55.1	63.9	-0.180	58.4	60.9	-0.025
Warfarin	10.9	7.6	0.113	10.3	9.2	0.011
DOAC	34.0	28.5	0.120	31.3	29.9	0.014
Antiplatelet/equivalent						
None	77.9	69.9	0.184	75.0	75.0	0.000
Aspirin + P2Y12 antagonist	16.1	25.1	-0.224	19.5	19.5	0.000
OAC + P2Y12 antagonist or P2Y12	6.0	5.0	0.044	5.5	5.5	0.000

[1] Weighting only mildy increased variance, so that the effective sample sizes were only 10-11% smaller than the actual sample: for DAPA, effective sample size 249.5, mean weight 1.0, SD 0.378, range 0.25 to 2.05; for EMPA, effective sample size 267.8, mean weight 1.0, SD 0.358, range 0.1 to 2.25.

ACEi/AT1 – angiotensin converting enzyme inhibitor/angiotensin receptor type 1; ACS/AMI – acute coronary syndrome/acute myocardial infarction; BMI – body mass index; CCB – calcium channel blocker; COPD – chronic obstructive pulmonary disease; CRT – cardiac resynchronization therapy; CVI/TIA – cerebrovascular insult/transitory ischemic attack; DOAC – direct oral anticoagulants; DVT/PE – deep vein thrombosis/pulmonary embolism; eGFR – estimated glomerular filtration rate; ER – emergency room visit; GLP1 – glucagon-like polypeptide 1; HF p/mr/r EF – heart failure with preserved/mildly reduced /reduced ejection fraction; HHF – hospitalization for heart failure; ICD – implantable cardioverter defibrillator; LVEF – left ventricular ejection fraction; MRA – mineralocorticoid receptor antagonist; NT-proBNP – N-terminal pro-brain natriuretic polypeptide; NYHA – New York Heart Association; OAC – oral anticoagulant

Table B3. Characteristics of patients with reduced left ventricular ejection fraction (LVEF ≤40%, HFrEF) by sodium-glucose transporter 2 inhibitor (SGLT2i) – dapagliflozin (DAPA) or empagliflozin (EMPA) - before and after optimization weighting. Data before weighting are raw data, data after weighting are weighted data: percentages, mean±standard deviation, or geometric mean (% coefficient of variation) for ln-transformed variables. Standardized mean differences (d)<0.1 indicate adequate balance between DAPA and EMPA-treated subjects.

	Before weighting (raw data)			After weighting (weighted data)[1]		
	DAPA	EMPA	d	DAPA	EMPA	d
N	359	229	---	359	229	---
Age (years)	67±12	66±12	0.135	67±12	67±12	0.000
SGLT2i start lagged (Study 1)		162 (70.7)	0.000	70.8	70.8	0.000
SGLT2i start early (Study 2)	105 (29.3)	67 (29.3)	0.000	29.3	29.3	0.000
Male	260 (72.4)	180 (78.6)	-0.062	73.8	75.3	-0.020
Chronic kidney disease	146 (40.7)	80 (34.9)	0.057	38.4	38.4	0.000
Preserved renal function	213 (59.3)	149 (65.1)	-0.057	61.6	61.6	0.000
LVEF (%)	30.8±6.9	32.6±6.3	-0.272	31.4±6.7	31.7±6.7	-0.050
NYHA 1	15 (4.2)	11 (4.8)	-0.006	4.4	4.4	0.000
NYHA 2	153 (42.6)	98 (42.8)	-0.002	42.7	42.7	0.000
NYHA 3	158 (44.0)	103 (45.0)	-0.010	44.4	44.4	0.000
NYHA 4	33 (9.2)	17 (7.4)	0.018	8.5	8.5	0.000
Ln(NT-proBNP)	3456 (205)	2771 (175)	0.178	3210 (207)	3141 (177)	0.017
Ln(BMI) (kg/m^2)	28.3 (17.9)	29.2 (17.0)	-0.167	28.6 (18)	28.7 (17)	-0.025
eGFR (mL/min/1.73m^2)	65.2±23.8	69.6±18.4	-0.191	66.5±23.9	67.4±22.5	-0.038
Diabetes mellitus						
No	80 (22.3)	59 (25.8)	-0.035	23.6	23.6	0.000
Yes	134 (37.3)	97 (42.4)	-0.050	39.3	39.3	0.000
Prediabetes	145 (40.4)	73 (31.9)	0.085	37.1	37.1	0.000
Hypertension	294 (81.9)	192 (83.8)	-0.020	82.5	83.2	-0.007
Dyslipidemia	249 (69.4)	179 (78.2)	-0.088	71.3	74.3	-0.030
Atrial fibrillation	154 (42.9)	92 (40.2)	0.027	41.8	41.8	0.000
Hemoglobin (g/L)	136±18	138±18	-0.097	137±18	137±18	-0.018
Anemic	122 (34.0)	66 (28.8)	0.052	32.5	31.5	0.010
Coronary artery disease	192 (53.5)	141 (61.6)	-0.081	56.1	57.1	-0.010
ACS/AMI over 12 months	6 (1.7)	3 (1.3)	0.004	1.7	1.3	0.004
ACS/AMI at start of SGLT2i	15 (4.2)	19 (8.3)	-0.041	5.3	6.3	-0.010
Carotide/peripheral artery disease	99 (27.6)	56 (24.5)	0.031	27.5	25.3	0.022
History of CVI/TIA	48 (13.4)	20 (8.7)	0.046	12.6	10.3	0.023
CVI/TIA at start of SGLT2i	3 (0.8)	2 (0.9)	0.000	0.7	1.1	-0.004
Peripheral artery disease	64 (17.8)	43 (18.8)	-0.010	18.3	17.9	0.004
COPD /asthma	44 (12.3)	21 (9.2)	0.031	11.7	9.8	0.019
HHF / 12 months or at start	223 (62.1)	117 (51.1)	0.110	59.1	56.6	0.025
0	136 (37.9)	112 (48.9)	-0.110	40.9	43.4	-0.025
1	174 (48.5)	100 (43.7)	0.048	46.6	47.7	-0.011
2	39 (10.9)	16 (7.0)	0.039	9.9	8.1	0.018
3	8 (2.2)	1 (0.4)	0.018	2.1	0.8	0.013
4	2 (0.6)	0	0.006	0.4	0	0.004
ER HF/ 12 months or at start	175 (48.7)	91 (39.7)	0.090	46.5	44.0	0.025
0	184 (51.3)	138 (60.3)	-0.090	53.5	56.0	-0.025
1	125 (34.8)	89 (34.9)	-0.001	34.4	36.1	-0.017
2	41 (11.4)	11 (4.8)	0.066	10.1	7.9	0.022
3	6 (1.7)	0	0.017	1.4	0	0.014
4	3 (0.8)	0	0.008	0.6	0	0.006
HHF at start of SGLT2i	137 (38.2)	79 (34.5)	0.034	36.8	37.4	-0.005
CRT or ICD or pacemaker	99 (27.6)	70 (30.6)	-0.030	27.6	30.0	-0.024
ACEi or AT1 antagonist	183 (51.0)	111 (48.5)	0.025	49.8	50.5	-0.008
Sacubitril-valsartan	149 (41.5)	109 (47.6)	-0.061	42.9	44.9	-0.020
MRA	337 (93.9)	211 (92.1)	0.017	94.1	92.2	0.019
Diuretic	304 (84.7)	175 (76.4)	0.083	82.5	80.5	0.020

Beta-blocker	336 (93.6)	217 (94.8)	-0.012	93.7	94.4	-0.008
CCB	50 (13.9)	31 (13.5)	0.004	14.8	12.8	0.019
Dyslipidemia treatment	249 (69.4)	171 (74.7)	-0.053	71.0	71.9	-0.009
None	110 (30.6)	58 (25.3)	0.053	29.0	28.1	0.009
High-potency statin	230 (64.1)	156 (68.1)	-0.041	65.2	66.1	-0.010
Statin + ezetimib	19 (5.3)	15 (6.6)	-0.013	5.8	5.8	0.000
Antidiabetic treatment	87 (24.2)	77 (33.6)	-0.094	26.9	28.3	-0.013
Metformin	59 (16.4)	61 (26.6)	-0.102	19.4	21.4	-0.020
GLP-1 agonist	19 (5.3)	26 (11.4)	-0.061	6.7	8.2	-0.015
Insulin	16 (4.5)	16 (7.0)	-0.025	4.4	6.1	-0.017
Other	21 (5.9)	9 (3.9)	0.019	5.7	3.8	0.019
Anticoagulation	170 (47.3)	99 (43.2)	0.041	46.6	46.3	0.003
None	189 (52.7)	130 (56.8)	-0.041	53.4	53.7	-0.003
Warfarin	38 (10.6)	26 (11.4)	-0.008	10.7	12.1	-0.014
DOAC	132 (36.8)	73 (31-9)	0.049	35.9	34.2	0.017
Antiplatelet/equivalent	84 (23.4)	62 (27.1)	-0.037	25.4	24.1	0.014
None	275 (76.6)	167 (72.9)	0.037	74.6	75.9	-0.014
Aspirin + P2Y12 antagonist	57 (15.9)	47 (20.5)	-0.047	17.5	18.2	-0.006
OAC + P2Y12 antagonist or P2Y12	27 (7.5)	15 (6.6)	0.010	7.9	5.9	0.020

[1] Weighting only mildy increased variance, so that the effective sample sizes were only around 3%-10% smaller than the actual sample: for DAPA, effective sample size 345.6, mean weight 1.0, SD 0.197, range 0.51 to 1.55; for EMPA, effective sample size 203.9, mean weight 1.0, SD 0.352, range 0.18 to 2.29.

ACEi/AT1 – angiotensin converting enzyme inhibitor/angiotensin receptor type 1; ACS/AMI – acute coronary syndrome/acute myocardial infarction; BMI – body mass index; CCB – calcium channel blocker; COPD – chronic obstructive pulmonary disease; CRT – cardiac resynchronization therapy; CVI/TIA – cerebrovascular insult/transitory ischemic attack; DOAC – direct oral anticoagulants; DVT/PE – deep vein thrombosis/pulmonary embolism; eGFR – estimated glomerular filtration rate; ER – emergency room visit; GLP1 – glucagon-like polypeptide 1; HHF – hospitalization for heart failure; ICD – implantable cardioverter defibrillator; LVEF – left ventricular ejection fraction; MRA – mineralocorticoid receptor antagonist; NT-proBNP – N-terminal pro-brain natriuretic polypeptide; NYHA – New York Heart Association; OAC – oral anticoagulant

Table B4. Characteristics of patients with mildly reduced or preserved left ventricular ejection fraction (LVEF >40%, HFm/pEF) by sodium-glucose transporter 2 inhibitor (SGLT2i) – dapagliflozin (DAPA) or empagliflozin (EMPA) - before and after optimization weighting. Data before weighting are raw data, data after weighting are weighted data: percentages, mean±standard deviation, or geometric mean (% coefficient of variation) for ln-transformed variables. Standardized mean differences (d)<0.1 indicate adequate balance between DAPA and EMPA-treated subjects.

	Before weighting (raw data)			After weighting (weighted data)[1]		
	DAPA	EMPA	d	DAPA	EMPA	d
N	158	215	---	158	215	---
Age (years)	72±10	70±10	0.138	71±11	71±10	-0.020
SGLT2i start lagged (Study 1)	139 (88.0)	166 (77.2)	0.108	81.8	81.8	0.000
SGLT2i start early (Study 2)	19 (12.0)	49 (22.8)	-0.108	18.2	18.2	0.000
HF p EF	97 (61.4)	139 (64.6)	-0.032	63.3	63.3	0.000
HF mr EF	61 (38.6)	76 (35.4)	0.032	36.7	36.7	0.000
Male	86 (54.4)	121 (56.3)	-0.018	55.4	56.2	-0.007
Chronic kidney disease	86 (54.4)	62 (28.8)	0.256	39.7	39.7	0.000
Preserved renal function	72 (45.6)	153 (71.2)	-0.256	60.3	60.3	0.000
LVEF (%)	52.0±7.6	52.4±7.6	-0.063	52.1±7.7	52.3±7.6	-0.018
NYHA 1	13 (8.2)	16 (7.4)	0.008	7.8	7.8	0.000
NYHA 2	77 (48.7)	138 (64.2)	-0.155	57.6	57.6	0.000
NYHA 3	64 (40.5)	56 (26.1)	0.145	32.2	32.2	0.000
NYHA 4	4 (2.5)	5 (2.3)	0.002	2.4	2.4	0.000
Ln(NT-proBNP)	1893 (188)	1617 (162)	0.133	1654 (171)	1997 (175)	-0.075
Ln(BMI) (kg/m^2)	30.1 (20.0)	29.8 (20.5)	0.056	29.9 (20.0)	30.0 (20.5)	-0.033
eGFR (mL/min/1.73m^2)	57.5±23.9	68.9±19.2	-0.534	63.4±23.3	64.5±19.5	-0.051
Diabetes mellitus						
No	34 (21.5)	65 (30.2)	-0.087	25.5	27.5	-0.020
Yes	84 (53.2)	91 (42.3)	0.108	47.8	45.9	0.018
Prediabetes	40 (25.3)	59 (27.4)	-0.021	26.7	26.5	0.002
Hypertension	147 (93.0)	203 (94.4)	-0.014	92.6	95.1	-0.025
Dyslipidemia	110 (69.6)	151 (70.2)	-0.006	69.7	70.0	-0.003
Atrial fibrillation	86 (54.4)	104 (48.4)	0.061	50.9	50.9	0.000
Hemoglobin (g/L)	131±18	133±18	-0.092	132±17	131±19	0.050
Anemic	64 (40.5)	73 (34.0)	0.066	38.0	36.7	0.013
Coronary artery disease	69 (43.7)	105 (48.8)	-0.052	45.9	46.2	-0.003
ACS/AMI over 12 months	2 (1.3)	2 (0.9)	0.003	1.0	0.7	0.003
ACS/AMI at start of SGLT2i	7 (4.4)	19 (8.8)	-0.044	5.5	7.7	-0.022
Carotide/peripheral artery disease	42 (26.6)	43 (20.0)	0.066	23.6	22.0	0.016
CVI/TIA at start of SGLT2i	0	1 (0.5)	-0.005	0	0.4	-0.004
Peripheral artery disease	37 (23.4)	37 (17.2)	0.062	20.2	19.2	0.010
COPD /asthma	28 (17.7)	24 (11.2)	0.066	15.0	12.9	0.021
HHF / 12 months or at start	71 (44.9)	72 (33.5)	0.115	39.1	36.7	0.024
0	87 (55.1)	143 (66.5)	-0.115	60.9	63.3	-0.024
1	57 (36.1)	58 (27.0)	0.091	31.6	28.6	0.030
2	12 (7.6)	11 (5.1)	0.025	6.3	5.9	0.003
3	2 (1.3)	3 (1.4)	-0.001	1.2	2.2	-0.009
ER HF/ 12 months or at start	71 (44.9)	84 (39.1)	0.059	39.8	42.9	-0.031
0	87 (55.1)	131 (60.9)	-0.059	60.2	57.1	0.031
1	58 (36.7)	66 (30.7)	0.060	33.5	32.9	0.006
2	12 (7.6)	17 (7.9)	-0.003	5.8	9.2	-0.034
3	1 (0.6)	1 (0.5)	0.002	0.5	0.8	-0.003
HHF at start of SGLT2i	51 (32.3)	45 (20.9)	0.113	26.4	24.0	0.026
CRT or ICD or pacemaker	18 (11.4)	19 (8.8)	0.026	10.4	10.8	-0.004
ACEi or AT1 antagonist	125 (79.1)	180 (83.7)	-0.046	80.4	83.3	-0.028
Sacubitril-valsartan	8 (5.1)	8 (3.7)	0.013	5.0	4.3	0.007

MRA	95 (60.1)	140 (65.1)	-0.050	61.7	64.3	-0.025
Diuretic	118 (74.7)	129 (60.0)	0.147	68.7	65.4	0.033
Beta-blocker	135 (85.4)	186 (86.5)	-0.011	86.3	86.5	-0.001
CCB	55 (34.8)	64 (29.8)	0.050	35.1	31.4	0.037
Dyslipidemia treatment						
None	59 (37.3)	63 (29.3)	0.080	33.2	31.5	0.017
High-potency statin	89 (56.3)	136 (63.3)	-0.069	60.0	61.6	-0.015
Statin + ezetimib	10 (6.3)	16 (7.4)	-0.011	6.8	6.9	-0.001
Antidiabetic treatment	66 (41.8)	72 (33.5)	0.083	37.8	35.7	0.022
Metformin	40 (25.3)	60 (27.9)	-0.026	23.4	30.5	-0.071
GLP-1 agonist	18 (11.4)	29 (13.6)	-0.021	10.4	14.9	-0.045
Insulin	14 (8.9)	8 (3.7)	0.051	7.8	3.6	0.042
Other	12 (7.6)	14 (6.5)	0.011	5.2	6.1	-0.009
Anticoagulation	93 (58.9)	105 (48.8)	0.100	54.4	52.8	0.016
None	65 (41.1)	110 (51.2)	-0.100	45.6	47.2	-0.016
Warfarin	22 (13.9)	16 (7.4)	0.065	13.5	8.8	0.047
DOAC	71 (44.9)	89 (41.4)	0.035	40.9	43.9	-0.030
Antiplatelet/equivalent	25 (15.8)	58 (27.0)	-0.112	21.0	23.5	-0.025
None	133 (84.2)	157 (73.0)	0.112	79.0	76.5	0.025
Aspirin + P2Y12 antagonist	17 (10.8)	46 (21.4)	-0.106	16.1	17.7	-0.017
OAC + P2Y12 antagonist or P2Y12	8 (5.1)	12 (5.6)	-0.005	4.9	5.8	-0.009

[1] Weighting midly-moderately increased the variance, so that the effective samples sizes were around 10-12% smaller than the actual sample: for DAPA, effective sample size 135.0, mean weight 1.0, SD 0.415, range 0.13 to 2.31; for EMPA, effective sample size 191.0, mean 1.0, SD 0.355, range 0.71 to 1.82.

ACEi/AT1 – angiotensin converting enzyme inhibitor/angiotensin receptor type 1; ACS/AMI – acute coronary syndrome/acute myocardial infarction; BMI – body mass index; CCB – calcium channel blocker; COPD – chronic obstructive pulmonary disease; CRT – cardiac resynchronization therapy; CVI/TIA – cerebrovascular insult/transitory ischemic attack; DOAC – direct oral anticoagulants; DVT/PE – deep vein thrombosis/pulmonary embolism; eGFR – estimated glomerular filtration rate; ER – emergency room visit; GLP1 – glucagon-like polypeptide 1; HHF – hospitalization for heart failure; HF mr/p EF – heart failure with mildly reduced/preserved ejection fraction; ICD – implantable cardioverter defibrillator; LVEF – left ventricular ejection fraction; MRA – mineralocorticoid receptor antagonist; NT-proBNP – N-terminal pro-brain natriuretic polypeptide; NYHA – New York Heart Association; OAC – oral anticoagulant
